# Molecular interplay between peptidoglycan integrity and outer membrane asymmetry in maintaining cell envelope homeostasis

**DOI:** 10.1128/jb.00331-25

**Published:** 2025-09-18

**Authors:** Sinjini Nandy, Arshya F. Tehrani, Augusto C. Hunt-Serracin, Jacob Biboy, Christine Pybus, Waldemar Vollmer, Joseph M. Boll

**Affiliations:** 1 Department of Biological Sciences, The University of Texas at Dallas 12335, Richardson, Texas, USA; 2 Centre for Bacterial Cell Biology, Newcastle University 5994, Newcastle upon Tyne, England, United Kingdom; 3 Institute for Molecular Bioscience, University of Queensland 1974, Brisbane, Queensland, Australia; Geisel School of Medicine at Dartmouth, Hanover, New Hampshire, USA

**Keywords:** LD-transpeptidase, peptidoglycan, maintenance of lipid asymmetry, gram-negative bacteria, cell envelope

## Abstract

The bacterial cell envelope is a critical interface with the environment, particularly in Gram-negative species where the outer membrane (OM) and peptidoglycan (PG) layers coordinate to maintain structural integrity and resist turgor. Although this coordination is essential for survival, the molecular mechanisms linking OM and PG homeostasis remain poorly understood. LD-transpeptidases (LDTs) are enzymes that crosslink peptides in PG and incorporate d-amino acids, but their physiological roles are not fully defined. Here, we characterize the activity of the LDT enzyme LdtJ in *Acinetobacter baumannii* and investigate the consequences of its deletion. Loss of LdtJ disrupts cell morphology, downregulates PG precursor genes (e.g., *dadA* and *alr*), and activates the stringent response, including elevated ppGpp levels and *dksA* upregulation. These defects are fully suppressed in a ∆*ldtJ* ∆*mla* double mutant, implicating the OM lipid transport Mla pathway in compensatory regulation. RNA sequencing revealed that transcriptional changes in the ∆*ldtJ* mutant are reversed in the double mutant, highlighting a functional interplay between PG remodeling and OM lipid asymmetry. Our findings suggest that LdtJ contributes to envelope integrity not only through PG modification but also by influencing broader regulatory and metabolic networks.

**IMPORTANCE:**

*Acinetobacter baumannii* is a leading cause of hospital-acquired infections and is highly resistant to antibiotics. Its survival relies on the integrity of the cell envelope, composed of the peptidoglycan (PG) layer and outer membrane (OM). While LD-transpeptidases are traditionally known for reinforcing PG structure through non-canonical crosslinking, our findings reveal that the LdtJ enzyme also plays a critical role in regulating cellular metabolism and stress responses. Deletion of *ldtJ* results in pronounced growth defects and abnormal cell morphology—phenotypes that are fully suppressed by disrupting the OM lipid asymmetry transport system, Mla. This genetic interaction uncovers a previously unrecognized link between PG remodeling and OM lipid homeostasis. These insights deepen our understanding of envelope coordination in Gram-negative bacteria.

## INTRODUCTION

The Gram-negative bacterial cell envelope is composed of three distinct layers: the inner (cytoplasmic) membrane, the peptidoglycan (PG) layer located in the periplasm, and the outer membrane (OM). The PG is a mesh-like polymer that maintains cell shape, resists osmotic pressure, and protects against environmental stressors (1, 2). Coordination between the PG and OM is essential for preserving envelope integrity and bacterial viability. Understanding how these layers interact and respond to stress is key to elucidating mechanisms of bacterial survival and morphology. PG is composed of glycan chains made of repeating N-acetylglucosamine (Glc*N*Ac)–N-acetylmuramic acid (Mur*N*Ac) disaccharides, connected by short peptides. In many Gram-negative bacteria, these peptides typically include l-alanine, d-glutamate, *meso*-diaminopimelic acid (*m*DAP), and two d-alanine residues, with the peptide covalently linked to Mur*N*Ac (3–5). PG biosynthesis and remodeling requires a suite of enzymes, including glycosyltransferases and transpeptidases for PG polymerization and cross-linkage, and PG hydrolases—such as endopeptidases, carboxypeptidases, amidases, lytic transglycosylases, and lysozymes—for maturation, turnover, and degradation (6–9).

Among enzymes involved in PG biosynthesis, penicillin-binding proteins (PBPs) play a crucial role. Class A and B PBPs possess DD-transpeptidase activity (DD-TPase), which forms 4-3 peptide crosslinks. These 4-3 crosslinks, formed from the fourth d-alanine of the donor pentapeptide moiety to the third *m*DAP of the acceptor stem (6, 10), are the most abundant, comprising 60–100% of total PG crosslinks during growth (depending on species) (1, 3). In contrast, LD-transpeptidases (LDTs) generate LD-crosslinks, also called 3-3 crosslinks, by linking the third *m*DAP residues of adjacent peptide stems (11–13). Unlike PBPs, LDTs utilize tetrapeptides as donor substrates, which are generated by DD-carboxypeptidases (DD-CPases) that remove the terminal d-alanine from pentapeptides. While 4-3 crosslinks dominate during exponential growth in *Escherichia coli*, LD-crosslinks typically comprising ~10% of total crosslinks during exponential growth increase to 16% in the stationary phase (14) and can reach up to 30–40% under certain OM stress conditions (15).

Although LDTs are not essential for survival of most bacteria, they perform a variety of functions beyond PG crosslinking (12). These include d-amino acid (DAA) incorporation into PG (16), maintenance of cell envelope integrity during lipopolysaccharide (LPS) transport defects (15), β-lactam resistance (17, 18), cold shock (19), and autolysin regulation (7). In *A. baumannii*, LdtJ is the only periplasmic LDT (20, 21), suggesting it catalyzes LD-crosslink formation and DAA incorporation, particularly during the stationary phase. A previous study (21) showed that deletion of *ldtJ* leads to reduced growth and altered cell morphology. While wild-type cells exhibited a coccobacillary shape, the ∆*ldtJ* mutant appeared coccoid and significantly shorter cell length, a phenotype that could be fully complemented. Although LDTs are typically associated with stationary-phase functions, our prior work suggested that LdtJ also contributes to growth-phase physiology. However, the molecular mechanism underlying these roles remains unclear and merits further investigation.

The Gram-negative OM is an asymmetric barrier, with phospholipids enriched in the periplasmic inner leaflet and LPS/lipooligosaccharide (LOS) present in the surface-exposed outer leaflet. This lipid asymmetry is critical for the cell to maintain the OM barrier function that protects against many external agents such as detergents, antibiotics, or lysins, which can disrupt membrane integrity or cause cell lysis (22, 23). Under stress conditions, phospholipids can become mislocalized to the outer leaflet, compromising the asymmetry. To counteract this, Gram-negative bacteria employ mechanisms to restore lipid distribution and preserve OM integrity (22, 24). In *A. baumannii*, two conserved systems help to maintain OM asymmetry: the Mla (maintenance of OM lipid asymmetry) pathway and phospholipase A (PldA) (25). The Mla system consists of six proteins—MlaA, MlaB, MlaC, MlaD, MlaE, and MlaF—that work together to remove mislocalized phospholipids from the OM and transport them back to the inner membrane (22, 25). In *E. coli*, MlaA, an OM lipoprotein, interacts with OmpC to capture mislocalized phospholipids (26). MlaC then shuttles them to the inner membrane MlaFEDB complex, where MlaF provides ATPase-driven energy for transport (27–30). In the absence of a functional Mla pathway, phospholipids accumulate on the cell surface, disrupting membrane asymmetry and compromising barrier integrity. As a result, *mla* mutants exhibit hypersensitivity to membrane-disrupting agents such as SDS and EDTA, which destabilize the OM by chelating divalent cations and disrupting lipid packing (22, 25).

The PG has long been considered the primary structural component of the bacterial cell envelope, providing mechanical strength and maintaining cell shape (5, 31). However, recent studies have shown that the LPS-enriched OM also plays a critical role in shaping cell morphology and reinforcing envelope integrity, particularly in resisting internal turgor changes (20, 32–34). Despite these advances, the molecular mechanisms that coordinate the functions of the PG layer and OM to maintain homeostasis remain poorly understood.

Herein, we characterized the enzymatic functions of LdtJ in *A. baumannii*. We confirmed its LDT and DAA incorporation activities and further identified LD-carboxypeptidase activity. Using the catalytically inactive mutant LdtJ_C390S_, we demonstrated that these enzymatic functions are not required for maintaining growth fitness or cellular morphology under standard conditions. While LdtJ was only thought to mediate LD-crosslinks and DAA incorporation during the stationary phase, our findings suggest it also actively contributes to PG synthesis and cellular fitness during active growth. Beyond PG structural remodeling, LdtJ may influence envelope-associated stress responses and metabolic pathways, pointing to a broader regulatory role in maintaining cellular homeostasis—identified for the first time in *A. baumannii*. Notably, we found that introducing *ldtJ* deletion into a strain lacking the Mla pathway—thereby disrupting OM lipid asymmetry—restored growth-phase defects, including impaired viability and altered morphology. These results underscore the importance of coordinated regulation between PG biosynthesis and OM homeostasis. Taken together, our findings reveal a functional interplay between PG integrity and OM lipid asymmetry, revealing how crosstalk between these systems supports envelope stability in *A. baumannii*.

## RESULTS

### Activities of the LDT LdtJ

Previous research on LdtJ showed that the gene product contributed to *A. baumannii* fitness and morphology (21). The Δ*ldtJ* mutant cells were spherical, unable to incorporate the fluorescent DAA, and had a growth defect relative to the wild-type strain. To expand on the previous report, we purified the wild-type enzyme and the catalytically inactive version (LdtJ_C390S_), in which the active-site cysteine was substituted with serine. Purification yielded two soluble protein products, as shown with Coomassie staining (Fig. S1A). Western blots with α-LdtJ-specific antisera (21) and an α-His antibody also showed there were two LdtJ protein products (Fig. S1B and C). MS analysis of the two excised bands showed the molecular weights (MWs) were 45.59 and 43.38 kDa (Fig. S1D). The MWs were consistent with the full-length LdtJ_His8X_ protein and a protein with a cleaved signal sequence between A21 and A22, consistent with di-alanine motif cleavage (35, 36). In general, cleavage of the di-alanine-containing signal sequence is associated with the successful export of proteins to the periplasm. However, the presence of both cleaved and uncleaved forms suggests that LdtJ is inefficiently exported in the *E. coli* strain BL21.

**Fig 1:**
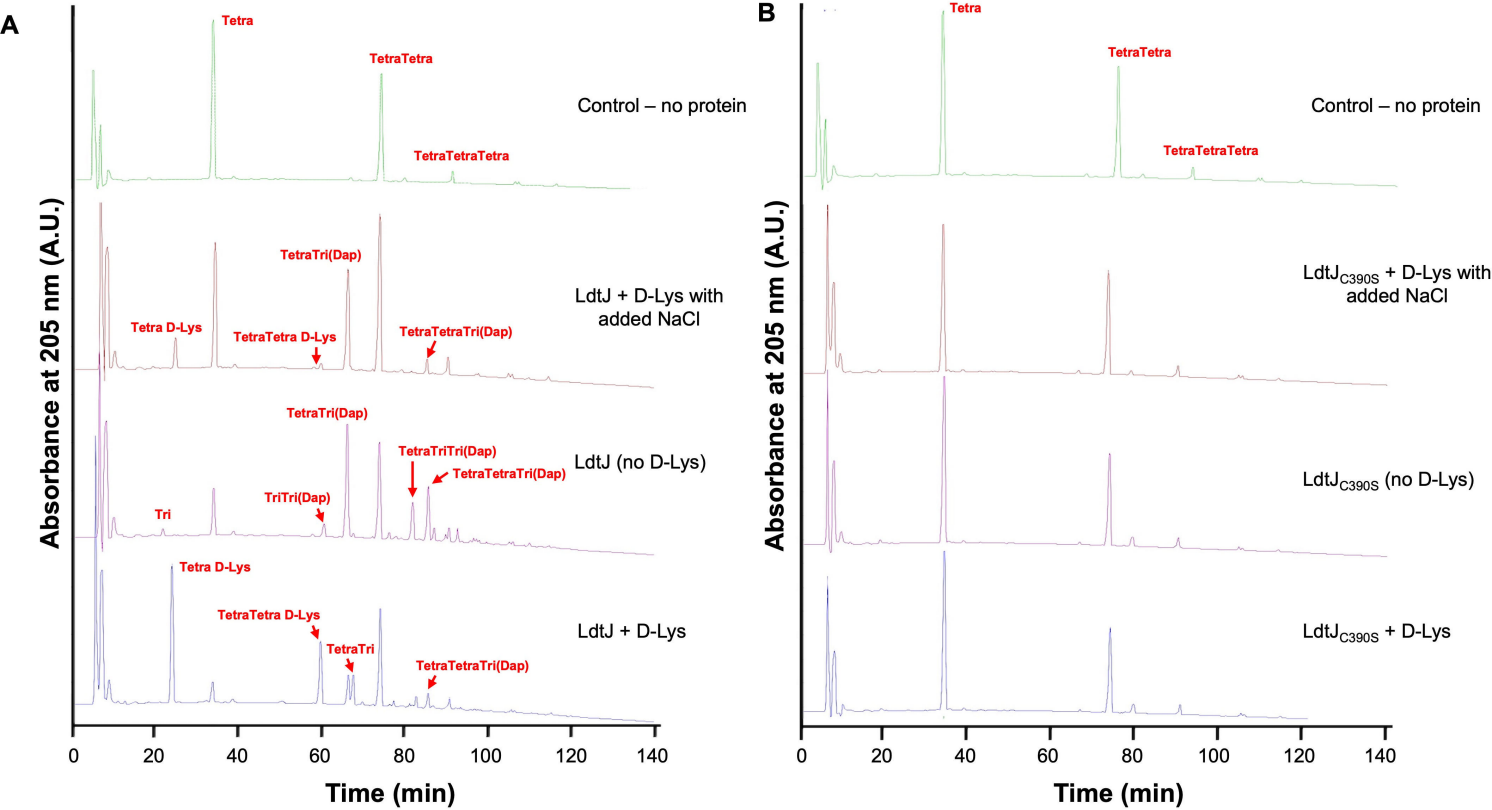
High-performance liquid chromatography (HPLC) traces of LdtJ enzymatic activity. (**A**) HPLC profiles of recombinant LdtJ incubated with purified peptidoglycan (PG) from *E. coli* BW25113Δ6LDT. Digestion with the muramidase cellosyl yields Tetra, TetraTetra, and TetraTetraTetra as the major monomeric, dimeric, and trimeric muropeptides (disaccharide peptide PG fragments). Reactions were performed under three conditions: with excess d-lysine (D-Lys), without d-Lys, and with d-Lys plus NaCl. (**B**) HPLC traces of the catalytically inactive mutant LdtJ_C390S_ under the same conditions. Muropeptide peaks are labeled accordingly. The observed profiles are consistent with LdtJ-mediated incorporation of d-amino acids (Tetra-d-Lys and TetraTetra-d-Lys), formation of LD-crosslinks (TriTri(Dap), TetraTri(Dap), TetraTriTri(Dap), and TetraTetraTri(Dap)), and low-level LD-carboxypeptidase activity (Tri, TriTri(Dap), and TetraTri). The absence of these modifications in the LdtJ_C390S_ mutant confirms the requirement of the catalytic cysteine for enzymatic function.

*In vitro* enzymatic assays with recombinant wild-type and active-site mutant enzymes were conducted using PG isolated from an *E. coli* BW25113Δ6LDT strain devoid of all LDT proteins (Fig. 1), as previously described (20). Wild-type LdtJ exhibited three distinct activities: LDT, DAA addition, and low-level LD-carboxypeptidase activities (Fig. 1A). In the absence of LdtJ, Tetra, TetraTetra, and TetraTetraTetra muropeptides were the main muropeptides released from the PG of *E. coli* BW25113Δ6LDT, consistent with previous PG analysis (18). LdtJ without the addition of d-lysine produced distinct LD-crosslinked TriTri(Dap), TetraTri(Dap), TetraTriTri(Dap), and TetraTetraTri(Dap), indicating LdtJ-mediated LD-LD-crosslinking. Additionally, we detected the muropeptides Tri and TetraTri, consistent with low-level LD-carboxypeptidase activity that removes the terminal d-alanine from tetrapeptides to generate tripeptides. Notably, the detection of TriTri(Dap) further supports dual LD-carboxypeptidase and LDT activities of LdtJ, as the formation of this species requires both the formation of LD-crosslinks forming TetraTri(Dap) and the trimming of its tetrapeptide to the tripeptide. Upon the addition of d-lysine, new peaks corresponding to Tetra-d-Lys and TetraTetra-d-Lys (d-Lys replacing d-Ala at position 4) were observed, indicating that LdtJ can incorporate DAAs into PG. However, in the presence of NaCl, the abundance of these d-Lys-containing muropeptides was significantly reduced compared to conditions without salt, suggesting that elevated ionic strength interferes with LdtJ-mediated DAA incorporation. Salt is known to modulate protein conformation and activity by altering electrostatic interactions (37). Under high-salt conditions, only the LD-crosslinked TetraTri(Dap) and TetraTetraTri(Dap) were detected, further indicating that d-Lys incorporation is selectively impaired. In contrast, LdtJ_C390S_ exhibited no detectable activity on PG (Fig. 1B). Collectively, these findings indicate that LdtJ possesses both LDT activity and low-level LD-carboxypeptidase activity, along with the ability to mediate d-lysine (DAA) incorporation into PG.

### Loss of LdtJ enzymatic activity has no impact on the fitness and morphology of *A. baumannii* during the growth phase

**Fig 2:**
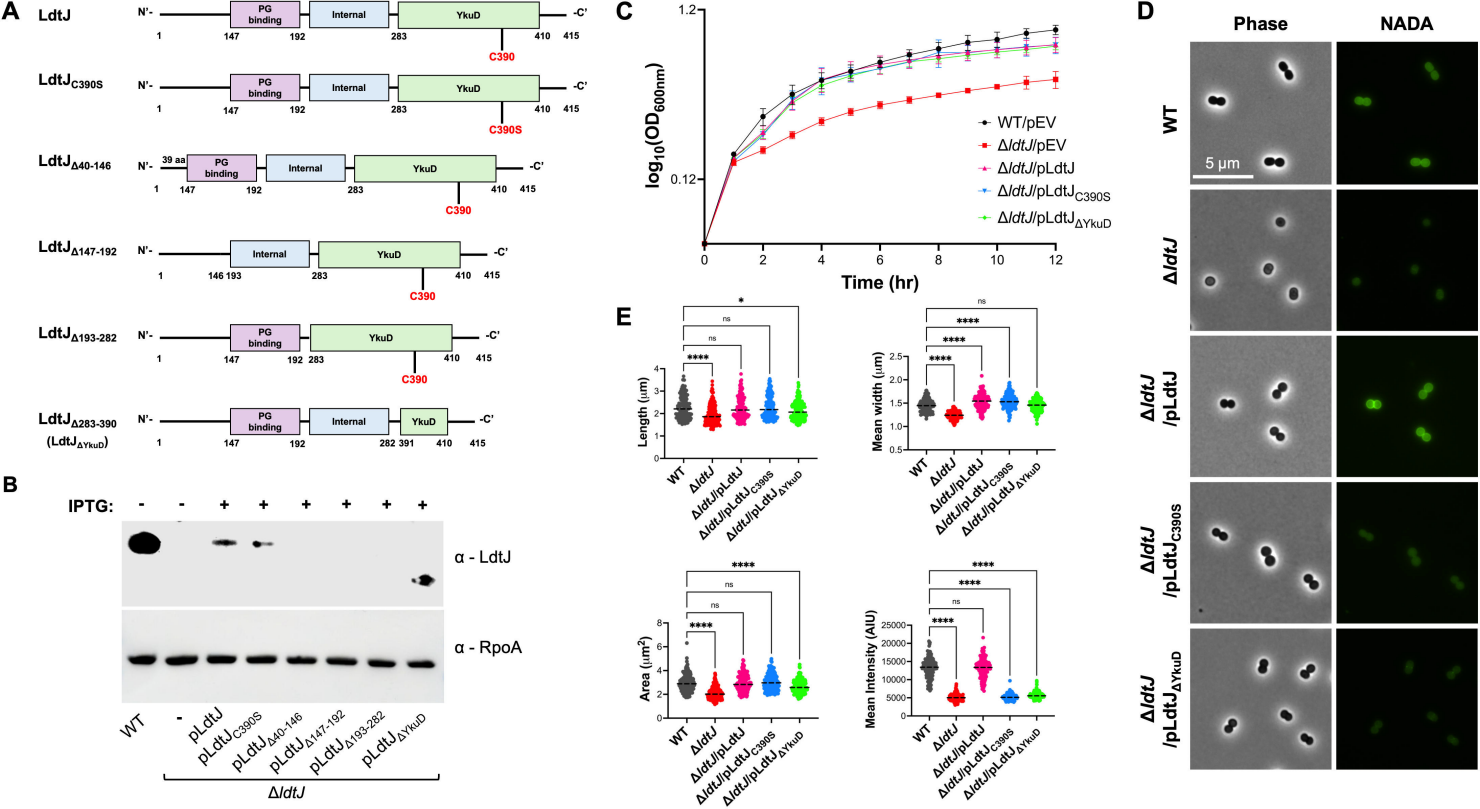
D**i**sruption of the enzymatic activity of LdtJ does not impair growth fitness or cell morphology. (**A**) Schematic representation of wild-type (WT) LdtJ and mutant constructs used in this study. (**B**) Western blot analysis using α-LdtJ antibody showing expression of LdtJ (44.5 kDa), LdtJ_C390S_ (44.49 kDa), and LdtJ_ΔYkuD_ (33.07 kDa). α-RpoA was used as a loading control. (**C**) Growth curve analysis of WT, ∆*ldtJ*, and complementation strains over 12 h in a 24-well plate (*n* = 3) (EV = empty vector). (**D**) Phase-contrast and NADA-stained microscopy images of WT, ∆*ldtJ*, and complementation strains. (**E**) Quantification of cell dimensions (length, width, and surface area) and intensity for WT, ∆*ldtJ*, and complementation strains (150 < *n* < 200 cells per strain). Each experiment was independently replicated three times, and one representative data set is shown. Statistical significance was determined using one-way ANOVA (*P < 0.05; ****P < 0.0001; ns = not significant).

The LDT, LdtJ, encodes four distinct domains following its signal peptide (Fig. 2A): (i) an N-terminal region (residues 1–146), (ii) a PG binding domain (147–192), (iii) an internal linker region (193–282), and (iv) the YkuD LDT domain (283–410), which includes the active site cysteine at position 390. To investigate the contribution of each domain in LdtJ stability, we generated a series of complementation constructs expressing LdtJ variants lacking individual domains. Western blot analysis (Fig. 2B) showed that deletion of the N-terminal region (LdtJ_Δ40–146_), PG binding domain (LdtJ_Δ147–192_) and the internal region (LdtJ_Δ193–282_), resulted in unstable proteins. In contrast, YkuD domain deletion (LdtJ_Δ283–390_/LdtJ_ΔYkuD_), despite removing the catalytic site, produced a stable protein, albeit at reduced levels relative to wild-type LdtJ. However, the expression levels were like those observed for wild-type LdtJ and the catalytically inactive mutant LdtJ_C390S_ used in complementation assays.

Based on a previously reported growth defect in the ∆*ldtJ* mutant (21), we hypothesized that the active site mutant LdtJ_C390S_ and LdtJ_ΔYkuD_ would impair bacterial fitness during growth. Consistent with prior findings, our analysis revealed a significant fitness defect in Δ*ldtJ* compared to the wild type (Fig. 2C), which was fully rescued by complementation restored to wild-type levels of the LdtJ allele. Surprisingly, complementation with either LdtJ_C390S_ or LdtJ_ΔYkuD_ fully restored fitness to wild-type levels, suggesting that the LDT, LD-carboxypeptidase, and DAA incorporation activities of LdtJ are not required for maintaining bacterial fitness under these conditions.

In addition to fitness defects, the Δ*ldtJ* strain exhibited altered cell morphology (Fig. 2D). Consistent with previous findings (21), wild-type cells displayed a coccobacillus shape, whereas Δ*ldtJ* appeared more coccoid. The morphological defect was rescued by complementation with the wild-type *ldtJ* allele, as well as with the LdtJ_C390S_ and LdtJ_ΔYkuD_ variants. Quantitative analysis confirmed that Δ*ldtJ* cells had significantly reduced cell dimensions, including shorter length, narrower width, and smaller surface area compared to the wild-type cells (Fig. 2E). The wild-type phenotype was fully restored upon complementation with the wild-type LdtJ and LdtJ_C390S_ alleles. The phenotype was partially restored when the LdtJ_ΔYkuD_ variant was expressed. As expected, fluorescence intensity of NADA (a fluorescent DAA) was not restored in strains complemented with LdtJ_C390S_ and LdtJ_ΔYkuD_.

These results indicate that LdtJ is essential for maintaining both bacterial fitness and proper cell morphology. Although the YkuD domain, which contains the active site, is required for LdtJ enzymatic activities, its inactivation does not compromise protein stability, bacterial fitness, or cell shape. This suggests that LdtJ contributes to these cellular functions through a mechanism independent of its catalytic activity.

### Disruption of the maintenance of the lipid asymmetry (Mla) pathway compensates for the growth and morphological defects observed in the Δ*ldtJ* mutant

**Fig 3:**
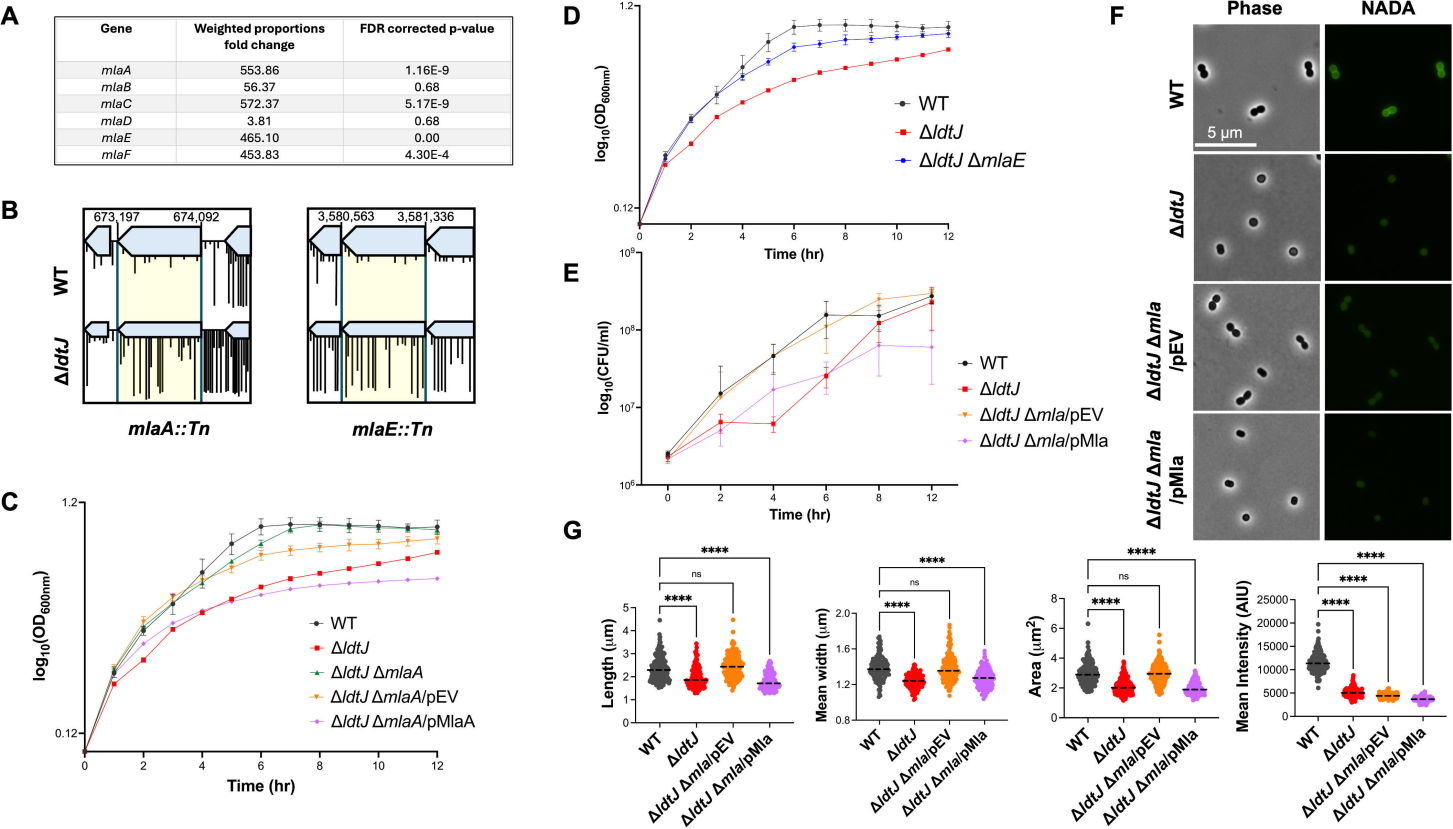
Disruption of the Mla pathway restores growth fitness, viability, and morphology in the Δ*ldtJ* mutant. (**A**) Transposon sequencing analysis of WT and Δ*ldtJ* strains. (**B**) Transposon insertion profiles for *mlaA::Tn* and *mlaE::Tn* in WT and Δ*ldtJ* backgrounds. (**C**) Growth curves of WT, Δ*ldtJ*, and Δ*ldtJ* Δ*mlaA* double mutants, with and without an empty vector, and with MlaA complementation. (**D**) Growth curves of WT, Δ*ldtJ*, and Δ*ldtJ* Δ*mlaE* strains. (**E**) Colony-forming unit (CFU) counts for WT, Δ*ldtJ*, Δ*ldtJ* Δ*mla* double mutants with empty vector, and with Mla complementation. (**F**) Phase-contrast and NADA-stained microscopy images of WT, Δ*ldtJ*, Δ*ldtJ* Δ*mla* double mutants with empty vector, and with Mla complementation. (**G**) Quantification of cell dimensions and fluorescence intensity from panel (F) (*n* = 150–200 cells). All experiments were independently replicated three times; one representative data set is shown. Statistical significance was determined using one-way ANOVA (****P < 0.0001; ns = not significant).

Given the severe growth defect in the ∆*ldtJ* mutant during the exponential growth phase, we sought to identify genetic suppressors that could restore this phenotype. To this end, we analyzed a previously published transposon mutagenesis data set comparing ∆*ldtJ* and wild-type strains (21, 38). This analysis revealed an enrichment of transposon insertions in all genes of the Mla pathway, with four genes showing statistically significant increases in insertion frequency in the ∆*ldtJ* background (Fig. 3A). We further compared specific transposon insertions in *mlaA::Tn* and *mlaE::Tn* in wild-type and ∆*ldtJ* strains (Fig. 3B). These findings led us to hypothesize that disruption of the Mla pathway suppresses the growth defect associated with the ∆*ldtJ* mutant.

To test this hypothesis, we constructed ∆*ldtJ* mutants in two ∆*mla* backgrounds. Both ∆*ldtJ* ∆*mlaA* and ∆*ldtJ* ∆*mlaE* strains restored growth fitness comparable to wild-type levels (Fig. 3C and D). Furthermore, complementation with MlaA in the ∆*ldtJ* ∆*mlaA* mutant (Fig. 3C) reverted the growth phenotype back to that of the ∆*ldtJ* single mutant, confirming that the suppression of the growth defect is dependent on the disruption of the Mla pathway.

Interestingly, SDS/EDTA susceptibility assays (Fig. S2A) revealed variability between independent ∆*ldtJ* ∆*mlaA* replicates. One replicate, derived from an SDS/EDTA-resistant ∆*mlaA1* parent, retained resistance, while the other, derived from an SDS/EDTA-sensitive ∆*mlaA2* parent, remained susceptible. Despite these differences in OM susceptibility, both ∆*ldtJ* ∆*mlaA* strains successfully restored the growth defect observed in the ∆*ldtJ* mutant (Fig. 3C**;** Fig. S2B). Moreover, complementation with MlaA in the SDS/EDTA-sensitive ∆*ldtJ* ∆*mlaA2* strain reverted the phenotype to that of the ∆*ldtJ* single mutant (Fig. S2B). These findings indicate that restoration of ∆*ldtJ* fitness is independent of OM integrity and instead likely results from disruption of lipid asymmetry. Whole-genome sequencing was performed on the wild-type, SDS/EDTA-resistant ∆*mlaA1* and SDS/EDTA-sensitive ∆*mlaA2*. The SDS/EDTA-sensitive strain had a mutation in a gene encoding a SAM-dependent methyltransferase [S72* (TCA→TAA)]. The resistant isolate did not carry any nucleotide variants compared to the wild type. Given the clean genetic background, we selected the resistant isolate for further analysis—hereafter referred to as ∆*ldtJ* ∆*mla*.

In addition to the Mla pathway, transposon mutagenesis also revealed a high frequency of transposon insertions in *pldA* (Fig. S2C), a gene encoding a phospholipase involved in maintaining OM lipid asymmetry. To determine whether *pldA* disruption could similarly suppress the ∆*ldtJ* growth defect, we generated the ∆*ldtJ* ∆*pldA* double mutant in a ∆*pldA* background. This strain fully restored growth fitness to wild-type levels (Fig. S2D). Moreover, the ∆*ldtJ* ∆*mla* ∆*pldA* triple mutant also rescued the ∆*ldtJ* growth defect, further supporting a link between OM asymmetry and suppression of the ∆*ldtJ* phenotype.

Beyond restoring growth, inactivation of Mla in the ∆*ldtJ* ∆*mla* mutant also corrected defects in cell viability (Fig. 3E) and morphology (Fig. 3F). The double mutant exhibited wild-type cell length, mean width, and surface area, fully rescuing the spherical morphology observed in ∆*ldtJ* cells (Fig. 3G). Taken together, these findings support the conclusion that disruption of OM lipid asymmetry—rather than general defects in membrane integrity—compensates for the fitness, viability, and morphological defects caused by loss of the LDT LdtJ.

### Transcriptomic profiling reveals compensatory gene expression changes in ∆*ldtJ* and ∆*ldtJ*∆*mla* mutants during growth

**Fig 4:**
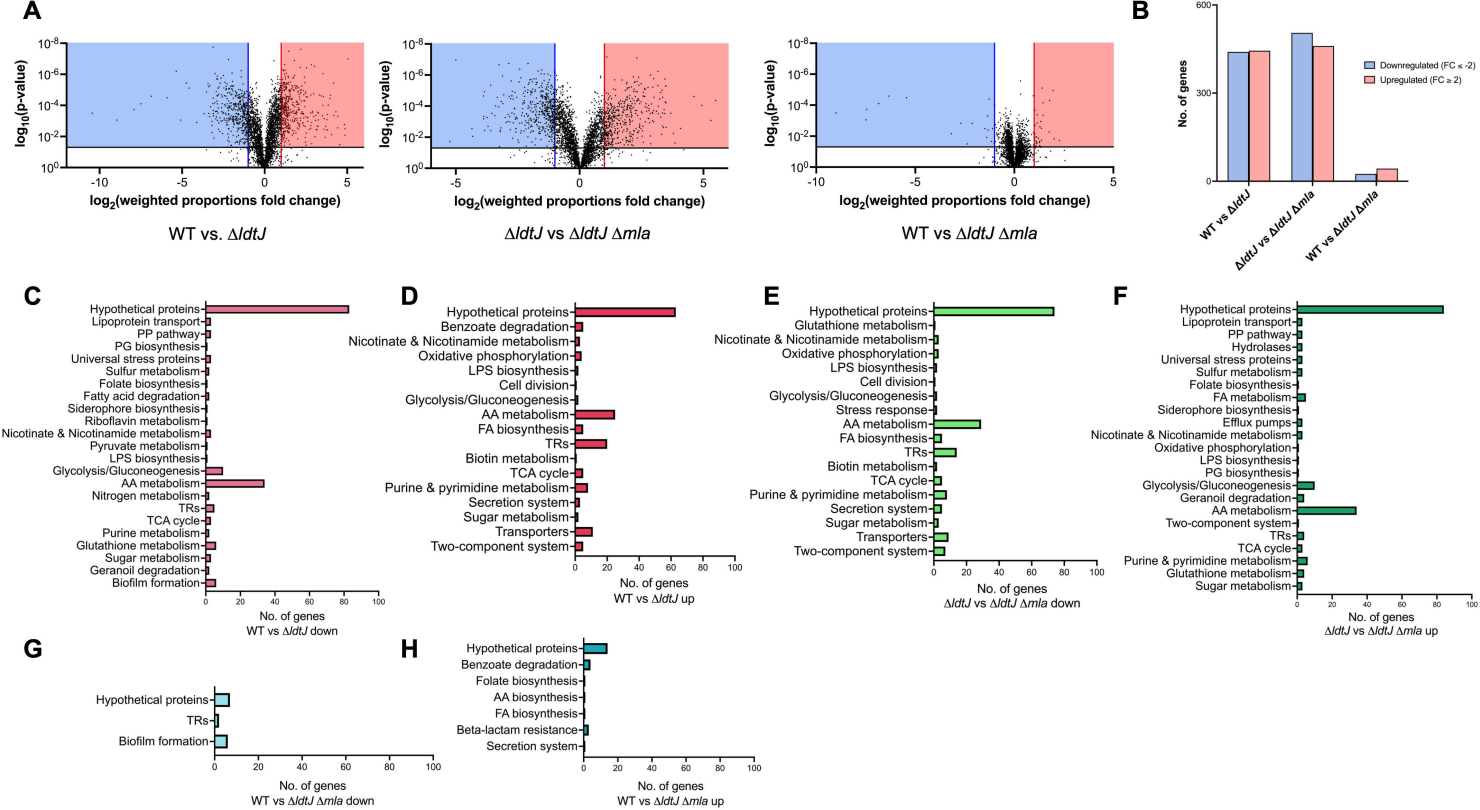
Differential gene expression profiles in WT, Δ*ldtJ*, and Δ*ldtJ* Δ*mla* strains. (**A**) Volcano plot showing differentially expressed genes during the growth phase. The blue box highlights significantly downregulated genes, while the red box highlights significantly upregulated genes. (**B**) Total number of significantly differentially expressed genes, defined by fold change ≥2 or ≤−2 and *P* < 0.05. (**C–H**) Number of differentially expressed genes involved in various biological pathways (e.g., cell envelope biogenesis and stress response) across the following comparisons: WT versus Δ*ldtJ*, Δ*ldtJ* versus Δ*ldtJ* Δ*mla*, and WT versus Δ*ldtJ* Δ*mla*. AA, amino acid; FA, fatty acid; LPS, lipopolysaccharide; PG, peptidoglycan; PP, pentose phosphate; TCA, tricarboxylic acid; TR, transcriptional regulator.

To investigate how disruption of the Mla pathway restores growth fitness, viability, and morphology in the ∆*ldtJ* mutant, we performed transcriptomic profiling during the logarithmic growth phase. RNA was isolated from wild-type, ∆*ldtJ,* and ∆*ldtJ* ∆*mla* strains, and gene expression profiles were compared. Differential expression analysis was conducted using a fold change threshold of ≥2 for upregulation or ≤−2 for downregulation, with a two-tailed *t*-test (*P* value < 0.05). Volcano plots comparing wild-type versus ∆*ldtJ* and ∆*ldtJ* versus ∆*ldtJ* ∆*mla* revealed similar patterns of differential gene expression between these two comparisons. In contrast, the wild-type versus ∆*ldtJ* ∆*mla* comparison showed fewer genes meeting the differential expression criteria, suggesting that Mla disruption partially restores the transcriptomic profile toward a wild-type state (Fig. 4A, G and H).

Compared to the wild-type, the ∆*ldtJ* mutant exhibited substantial transcriptomic changes, with 440 genes downregulated and 444 upregulated (Fig. 4B). In ∆*ldtJ* versus ∆*ldtJ* ∆*mla,* 505 genes were downregulated and 460 were upregulated, indicating a similarly broad shift in gene expression. In contrast, the ∆*ldtJ* ∆*mla* strain showed only 25 genes downregulated and 43 upregulated relative to the wild type. These results suggest that the transcriptomic profile of ∆*ldtJ* ∆*mla* closely resembles that of wild type, consistent with our phenotypic analyses that Mla disruption compensates for the loss of LdtJ.

Notably, 363 of the 440 genes downregulated in ∆*ldtJ* relative to wild type were upregulated in ∆*ldtJ* ∆*mla* compared to ∆*ldtJ* (Fig. 4C through F). These genes were enriched in pathways related to amino acid metabolism, fatty acid degradation, and biofilm formation. Conversely, 387 of the 444 genes upregulated in ∆*ldtJ* were downregulated in ∆*ldtJ* ∆*mla***,** including genes involved in nutrient transport and metabolism, fatty acid biosynthesis, stress response, fructose metabolism, and the bacterial secretion system. These reciprocal expression patterns suggest that Mla disruption rebalances key metabolic and stress response pathways disrupted by the loss of LdtJ.

The inverse gene expression patterns observed between wild-type versus ∆*ldtJ* and ∆*ldtJ* versus ∆*ldtJ* ∆*mla* suggest the involvement of a global regulatory mechanism. These findings indicate that the loss of *ldtJ* activates compensatory transcriptional programs, which are further modulated by disruption of the Mla pathway. This layered regulatory response likely underlies the restoration of fitness, viability, and morphology in the ∆*ldtJ* ∆*mla* mutant.

### Metabolic and regulatory disruptions in ∆*ldtJ* are mitigated by loss of Mla function during exponential growth

**Fig 5:**
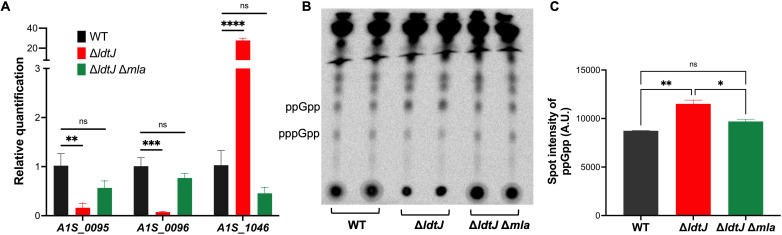
Reverse transcriptase quantitative PCR (RT-qPCR) analysis and ^32^P-radiolabeled thin-layer chromatography (TLC). (**A**) RT-qPCR analysis showing relative expression levels of *A1S_0095*, *A1S_0096*, and *A1S_1046* in WT, Δ*ldtJ*, and Δ*ldtJ* Δ*mla* strains. RT-qPCR experiments were independently replicated three times; one representative data set is shown. Statistical significance was determined using a two-tailed *t*-test (**P < 0.01; ***P < 0.001; ****P < 0.0001; ns = not significant). (**B**) TLC of radiolabeled (^32^P) lipid extracts from WT, Δ*ldtJ*, and Δ*ldtJ* Δ*mla* strains. ppGpp was identified based on migration using a 1.5 M KH_2_PO_4_ (pH 3.4) solvent system. (**C**) Quantification of TLC spot intensities. TLC experiments were independently replicated twice in duplicate; one representative data set is shown. Error bars represent standard deviations. Statistical significance was determined using one-way ANOVA (*P < 0.05; **P < 0.01; ns = not significant).

To validate the RNA-sequencing (RNA-seq) results, we performed reverse transcriptase quantitative PCR (RT-qPCR) on DNase-treated RNA samples from wild-type, ∆*ldtJ*, and ∆*ldtJ* ∆*mla* strains. Complementary DNA (cDNA) was synthesized and amplified using gene-specific primers targeting transcripts identified as differentially expressed in the RNA-seq data set. We selected *dadA*, *alr*, and *argO* for validation based on their significant expression changes and functional relevance*—dadA* and *alr* are associated with d-alanine metabolism and PG synthesis (39, 40), while *argO* encodes an arginine exporter involved in maintaining intracellular arginine homeostasis and modulating cellular stress responses (41). In the ∆*ldtJ* mutant, *dadA* (*A1S_0095*) and *alr* (*A1S_0096*) were significantly downregulated relative to wild type, with fold changes of 41.0 and 26.9, respectively (Table S1). Conversely, *argO* (*A1S_1046*) was markedly upregulated by 32.9-fold. Interestingly, in the ∆*ldtJ* versus ∆*ldtJ* ∆*mla* comparison (Table S2), an inverse expression pattern was observed: *dadA* and *alr* were upregulated by 45.1 and 39.6-fold, respectively, while *argO* expression was reduced by 31.9-fold compared to the ∆*ldtJ* mutant. These RT-qPCR results corroborated the RNA-seq data, confirming the significant downregulation of *dadA* and *alr* and upregulation of *argO* in the ∆*ldtJ* mutant compared to wild type (Fig. 5A). In the wild-type versus ∆*ldtJ* ∆*mla* comparison (Table S3), fold changes for *dadA*, *alr*, and *argO* were below the differential expression threshold indicating expression levels comparable to those in the wild-type background. These findings were consistent with RT-qPCR results, further supporting the conclusion that Mla disruption restores gene expression toward the wild-type state.

In addition to transcriptional validation, phenotypic confirmation of the RNA-seq results was obtained by examining the expression of *dksA* (*A1S_0248*), a transcriptional regulator involved in the bacterial stringent response. *dksA* expression was found to be upregulated by 2.8-fold in the ∆*ldtJ* mutant and downregulated by 3.1-fold in ∆*ldtJ* ∆*mla. dksA* modulates RNA polymerase activity and functions in concert with the alarmone (p)ppGpp—primarily guanosine-5′, 3′-tetraphosphate, ppGpp—to regulate the transcription of stress response genes, including those involved in PG remodeling under nutrient-limiting conditions (42). While DksA does not directly bind ppGpp, it is essential for mediating its effect on transcriptional reprogramming.

To investigate the functional link between *dksA* expression and the stringent response, we quantified intracellular stringent response nucleotide levels using radiolabeled phosphate (^32^P) followed by thin-layer chromatography (TLC), as previously described (43). TLC analysis revealed elevated accumulation of ppGpp in the ∆*ldtJ* mutant compared to both wild-type and ∆*ldtJ* ∆*mla* (Fig. 5B). Quantification of TLC spot intensities (Fig. 5C) confirmed this observation, indicating an enhanced stringent response in the ∆*ldtJ* background.

In summary, both RT-qPCR and phenotypic validation confirm that deletion of *ldtJ* disrupts the expression of genes associated with PG synthesis and activates stress response pathways. This is evidenced by altered expression of *dadA*, *alr*, *argO*, and *dksA*, along with elevated intracellular ppGpp levels. These disruptions reflect a broader imbalance in PG-related and unrelated metabolic and regulatory networks. Strikingly, additional deletion of *mla* in the ∆*ldtJ* ∆*mla* mutant restores transcriptional and physiological homeostasis, mitigating the stress phenotypes observed in the ∆*ldtJ* single mutant. Taken together, these findings reveal a functional interplay between OM lipid homeostasis and PG remodeling in *A. baumannii* and suggest that perturbations in one system can be compensated for adaptive changes in the other.

## DISCUSSION

In this study, we uncover a previously unrecognized functional interplay between PG remodeling and OM lipid homeostasis in *A. baumannii*, centered on the LDT LdtJ. Building on prior work linking *ldtJ* to cell shape and growth (21), we demonstrate that LdtJ contributes to both enzymatic and non-enzymatic functions essential for maintaining envelope integrity. Deletion of *ldtJ* resulted in severe growth and morphological defects, disrupted d-alanine metabolism, and activated stress responses, including elevated ppGpp levels. Remarkably, disruption of the Mla lipid transport system suppressed these phenotypes, revealing a compensatory relationship between PG remodeling and OM lipid asymmetry.

Biochemical assays confirmed that LdtJ catalyzes LD-PG crosslinking via *m*DAP and incorporates DAAs such as d-lysine, consistent with the activity of LDTs in *E. coli* and *Mycobacterium tuberculosis* (44, 45). LdtJ also exhibited low-level LD-carboxypeptidase activity, and the catalytic cysteine was essential for function, as shown by the loss-of-function phenotype in the LdtJ_C390S_ variant (46).

Mutagenesis and complementation analyses revealed that non-catalytic domains of LdtJ are critical for protein stability or folding, while deletion of the catalytic YkuD domain preserved growth and morphology. These findings also suggest that LdtJ has non-enzymatic roles, potentially serving as a structural or scaffolding protein. This dual-function model is supported by similar observations in *Francisella tularensis*, *Campylobacter jejuni*, and *Mycobacterium smegmatis*, where PG-modifying enzymes influence cell shape independently of catalytic activity (47–49). Notably, LdtJ includes a 146 amino acid N-terminal domain (Fig. 2A) that is not conserved in *E. coli* LdtE, which may confer additional functionality.

To investigate how Mla disruption suppresses ∆*ldtJ* defects, we constructed double and triple mutants. Loss of *mlaA* or *pldA* (both involved in OM lipid asymmetry) restored growth and morphology in strains lacking *ldtJ*. These effects were independent of OM integrity, implicating lipid asymmetry as the key modulator. These findings align with studies in *Vibrio cholerae*, where OM remodeling genes buffer defects caused by cell wall enzyme loss (50), and support broader models of envelope coordination (9, 51).

Transcriptomic profiling revealed widespread gene dysregulation in ∆*ldtJ*, including strong downregulation of *dadA* and *alr*—enzymes essential for d-alanine metabolism and PG biosynthesis (39, 40) and upregulation of *argO*, an arginine exporter linked to nitrogen stress (41). These changes likely disrupt PG precursor synthesis by altering glutamate and *m*DAP availability, contributing to envelope stress. Notably, these transcriptional imbalances were reversed in the ∆*ldtJ* ∆*mla* mutant, suggesting that Mla disruption restores metabolic homeostasis.

Further analysis revealed upregulation of stress-associated genes in ∆*ldtJ*, including *dksA*, and increased ppGpp accumulation, consistent with activation of the stringent response (42, 52). This response, likely triggered by amino acid and PG precursor imbalances, is known to reduce growth and cell size (53, 54), mirroring ∆*ldtJ* phenotypes. Restoration of *dksA*, *dadA*, *alr*, and *argO* expression in the double mutant underscores the regulatory link between OM lipid asymmetry and PG homeostasis.

∆*ldtJ* cells also upregulated a broader set of metabolic and transport-associated genes linked to stress adaptation, nitrogen balance, and PG precursor synthesis (Table S1). Some of them included amino acid transporters*—putP* (*A1S_1530*), *mgtA* (*A1S_2070*), catabolism*—aspQ* (*A1S_1466*), *aspC* (*A1S_2508*), biosynthesis*—lysC* (*A1S_1142*), *dat* (*A1S_2454*), *ddc* (*A1S_2453*), and a lysozyme-annotated gene (*A1S_2016*) potentially involved in PG turnover. Many of these genes are associated with nutrient salvage, redox balance, or polyamine metabolism and are commonly induced under envelope or nutritional stress (55–61). Strikingly, expression of these genes was downregulated in the ∆*ldtJ* ∆*mla* background, further supporting the role of Mla disruption in alleviating envelope stress and rebalancing cellular metabolism.

These findings suggest that the transcriptional signatures observed in ∆*ldtJ* may reflect impaired amino acid uptake or availability. Disrupting the Mla system likely alters OM lipid asymmetry in a way that relieves periplasmic or envelope stress (62), suppressing the activation of the stringent response in ∆*ldtJ* mutant. This may restore nutrient flux, rebalance metabolism, or prevent activation of stress sensors that otherwise trigger ppGpp accumulation and transcriptional dysregulation. Changes in OM lipid composition may also indirectly affect the biophysical environment of the periplasm (63), buffering the loss of LdtJ.

Although reduced growth rate (as observed in ∆*ldtJ*) can trigger some transcriptional changes, the observation that disruption of *mla* restores both growth and gene expression to near wild-type levels—alongside the complementation experiments—suggests that the transcriptome shift in ∆*ldtJ* is largely stress-specific, rather than growth rate-dependent. This highlights the tight integration of PG synthesis, amino acid metabolism, and OM homeostasis in *A. baumannii*, with the cell reprogramming gene expression to buffer envelope stress. LdtJ thus functions beyond PG crosslinking, contributing to envelope integrity and cellular homeostasis—a regulatory role not previously described in this organism.

Future studies should explore whether non-enzymatic LdtJ variants (e.g., LdtJ_C390S_ or LdtJ_ΔYkuD_) influence metabolic regulation and whether LdtJ physically interacts with other envelope-associated proteins. Protein-protein interaction studies to identify LdtJ interaction partners will be valuable in uncovering its enzymatic and non-enzymatic roles in PG remodeling. In parallel, muropeptide analysis of the catalytically inactive LdtJ variants may provide further insight into how specific domains contribute to PG architecture or signaling. Lipidomic analyses of the ∆*ldtJ* ∆*mla* double mutant will further clarify how changes in OM lipid composition might contribute to the observed suppression phenotype. Dissecting the mechanistic basis of the LdtJ-Mla interplay—whether through cross-regulation, envelope biophysics, or shared stress signaling—will further illuminate the compensatory dynamics between PG remodeling and OM lipid homeostasis.

This study reveals a novel link between LDT-mediated PG remodeling and OM lipid homeostasis in *A. baumannii*. Disruption of *ldtJ* activates envelope and metabolic stress responses, while loss of *mla* restores cellular balance. These findings advance our understanding of envelope coordination and suggest new strategies for targeting interconnected stress pathways in Gram-negative pathogens.

## MATERIALS AND METHODS

### Bacterial strains and growth

All strains and plasmids used in this study are listed in Table S4. All *A. baumannii* 17978 strains were initially cultured from −80°C stocks on Luria-Bertani (LB) agar at 37°C. When appropriate, kanamycin was used for selection at a final concentration of 25  µg/mL (Kan^25^).

### Construction of single, double, and triple genetic mutants

All *A. baumannii* mutations were generated as previously described (64). Briefly, the recombinase plasmid pAT03 (expressing REC_Ab_) was expressed in *A. baumannii* ATCC 17978. A linear PCR product containing the kanamycin resistance cassette FLP recombination target (FRT) sites and 125 bp regions of homology to *mlaA*, *mlaE*, and *pldA* was transformed into each strain to generate the respective single deletion mutants: Δ*mlaA*, Δ*mlaE*, and Δ*pldA*. To construct double mutants (Δ*ldtJ* Δ*mlaA*, Δ*ldtJ* Δ*mlaE*, and Δ*ldtJ* Δ*pldA*) and the triple mutant (Δ*ldtJ* Δ*mlaA* Δ*pldA*), the pAT03 was first introduced into the appropriate single or double mutant backgrounds. A second linear PCR product targeting *ldtJ*, also containing an FRT-flanked kanamycin cassette with 125 bp homology arms, was then transformed into these strains. Transformants were recovered in LB medium, pelleted by centrifugation, and plated on LB agar supplemented with Kan^25^ for selection. All mutations were verified by PCR and Sanger sequencing.

Following mutant construction, the pMMB67EH::REC_Ab_ plasmid was cured as previously described (64). The pMMB67EH::FLP recombinase was then introduced into the cured mutants. Cells were recovered in LB and plated on LB agar containing IPTG to induce FLP expression. Successful excision of the kanamycin cassette was confirmed by PCR.

### Construction of LdtJ_C390S_ and MlaA expression clones

The *ldtJ* coding sequence was amplified from *A. baumannii* ATCC 17978 gDNA. To clone in pMMB67EHKn, the PCR product and plasmid were digested with KpnI and SalI restriction enzymes. The reverse primer was designed to introduce a C390S point mutation, replacing the cysteine at position 390 with serine during amplification. The resulting construct LdtJ_C390S_ was confirmed by Sanger sequencing and transformed into the Δ*ldtJ* strain. This strain was used for Western blotting, complementation, and microscopic studies. For induction of LdtJ_C390S_ expression, culture was grown in Kan^25^ overnight in LB, followed by induction with 2 mM isopropyl-β-d-thiogalactopyranoside (IPTG) the next day.

The *mla* coding sequence was similarly amplified from *A. baumannii* ATCC 17978 gDNA and cloned into pMMB67EHKn using restriction enzymes EcoRI and KpnI. The construct was verified by sequencing and transformed into Δ*ldtJ* Δ*mlaA* double mutant. For overexpression studies, the strain was induced with 0.1 mM IPTG in the presence of Kan^25^.

### Construction of LdtJ domain deletion constructs

Four domain-deletion constructs of *ldtJ* were generated to investigate the functional role of specific regions. The first construct, LdtJ_Δ40–146_ contains the N-terminal 39 amino acids, including the predicted export sequence, while deleting residues 40–146 of the N-terminal domain. The remaining three constructs were designed as follows: (i) LdtJ_Δ147-192_; deletion of residues 147–192, LdtJ_Δ193–282_; deletion of residues 193–282 and LdtJ_ΔYkuD_; deletion of the conserved YkuD domain, retaining only the C-terminal 25 amino acids, which include the α-LdtJ binding region. All constructs were synthesized by Twist Bioscience. The DNA fragments were digested with KpnI and SalI and cloned into the pMMB67EHKn expression vector. Constructs were verified by Sanger sequencing and transformed into Δ*ldtJ* strain. Expression was induced with 2 mM IPTG, and the resulting strains were used for Western blotting, growth curve analysis, and morphology studies.

### Optical density growth curves

Overnight cultures were back-diluted to an initial OD_600_ 0.05 and distributed into a 24-well plate in triplicate biological replicates. For complementation strains, Kan^25^ and IPTG (2 mM or 0.1 mM, as appropriate) were used. Growth was measured using a BioTek SynergyNeo^2^ microplate reader, which recorded OD_600_ readings hourly at 37°C with continuous orbital shaking. Growth curves were plotted in GraphPad Prism 10 (version 8.4.1). Each experiment was independently replicated three times, and one representative data set was reported.

### CFU growth curve

Overnight cultures were initiated from a single colony and grown at 37°C in LB broth. Cultures were then back-diluted in triplicates to an initial OD_600_ 0.05 and incubated at 37°C with shaking. At designated time points, aliquots were collected, serially diluted, and plated on LB agar. For complementation and overexpression strains, Kan^25^ and IPTG (2 mM or 0.1 mM, as appropriate) were included in both the liquid and solid media. Plates were incubated overnight at 37°C, and the colony-forming units (CFUs) were enumerated. The viability curve was plotted using GraphPad Prism 10 (version 8.4.1). Each experiment was independently replicated three times, and one representative data set was reported.

### SDS/EDTA assay

Overnight cultures were patched onto standard LB agar plates and LB agar supplemented with 0.012% SDS and 0.185 mM EDTA to assess OM integrity. Plates were incubated overnight at 37°C. The following day, images were captured using a biomolecular imager (Amersham ImageQuant 800 by Cytiva).

### Fluorescent NADA staining

Overnight cultures were back-diluted to an OD_600_ 0.05 and grown at 37°C in LB broth until reaching mid-logarithmic growth phase. Complementation strains were grown in the presence of Kan^25^ and IPTG as required. The cells were harvested, washed once with LB, and resuspended in fresh LB. 2 µL of 10 mM NADA (NBD-(linezolid-7-nitrobenz-2-oxa-1,3-diazol-4-yl)-amino-d-alanine; Thermo Fisher Scientific) was added. The cells were incubated with NADA at 37°C for 20 min with shaking in the dark. Following incubation, the cells were washed once and fixed with 1× phosphate-buffered saline (PBS) containing a (1:10) solution of 16% paraformaldehyde.

### Microscopy

Fixed cells were immobilized on 1.5% agarose pads and imaged using an inverted Nikon Eclipse Ti-2 widefield epifluorescence microscope equipped with a Photometrics Prime 95B camera and a Plan Apo 100× 1.45-numerical-aperture lens objective. Green fluorescence images acquired using a filter cube with a 470/40 nm excitation filter and 535/50 emission filter. Image acquisition was performed using NIS Elements software (Nikon).

### Image analysis

All microscopy images were processed with Fiji. Quantitative analysis was performed with the MicrobeJ plugin. Parameters including cell length, mean width, area, and mean fluorescence intensity were measured in MicrobeJ. For each strain, at least 100 cells were analyzed per experiment. Data were plotted in GraphPad Prism 10 (version 8.4.1). Each experiment was independently replicated three times, with one representative data set reported in the quantification, and one representative image was included in the figure.

### Construction of LdtJ overexpression strains for protein purification

The *ldtJ* coding sequence was amplified from *A. baumannii* ATCC 17978 gDNA. The *ldtJ*_C390S_ variant was generated using a reverse primer designed to introduce the C390S point mutation and append a His_8X_ during PCR amplification. Both amplicons were cloned into the NdeI and BamHI restriction sites in pT7-7Kn expression vector. The resulting plasmids, pT7-7Kn::*ldtJ*_His8X_ and pT7-7Kn::*ldtJ*_C390S_*_-_*_His8X_, were transformed into chemically competent *E. coli* DH5α cells for propagation and sequence verification via Sanger sequencing. Verified constructs were transformed into chemically competent *E. coli* C2527 (BL21) cells (New England BioLabs, Inc.) for protein expression, purification, and Western blot analysis.

### Purification of recombinant LdtJ

*E. coli* BL21 cells carrying pT7-7Kn::*ldtJ*_His8X_ and pT7-7Kn::*ldtJ*_C390SHis8X_ were cultured in 1 and 2 L LB broth, respectively, and induced with 1 mM IPTG at 16°C overnight. The cells were harvested by centrifugation, washed in cold 1× PBS, pelleted, and stored at −80°C overnight. Frozen pellets were thawed on ice and resuspended in 20 mL lysis buffer (20 mM Tris, 300 mM NaCl, and 10 mM imidazole, pH 8). The cells were lysed by sonication (Fisher Scientific Model 120 Sonic Dismembrator) for 20 s on and off for a total of 10 cycles at 80% amplitude. Lysates were centrifuged at 15,428 × *g* for 10 min at 4°C. The supernatant was incubated with HisPur Ni-nitrilotriacetic acid (NTA) resin (Thermo Fisher Scientific) prewashed with lysis buffer, on a rotator for 2 h at 4°C. The mixture was loaded onto a 10 mL gravity-flow column (Thermo Fisher Scientific) and washed sequentially with 20 mL lysis buffer containing increasing concentrations of imidazole (0, 15, and 30  mM). Elution was performed with eight fractions using 500 µL of elution buffer (20 mM Tris, 300 mM NaCl, and 250 mM imidazole, pH 8), incubated with the column for 5  min before each gravity elution. Elution fractions containing protein (as determined by SDS-PAGE) were pooled and dialyzed overnight at 4°C using a 20 kDa MW CO 12 mL capacity dialysis cassette (Thermo Fisher Scientific) in dialysis buffer overnight (10 mM Tris, 50 mM KCl, 0.1 mM EDTA, and 5% glycerol, pH 8). Protein purity and identity were confirmed by Western blotting using both α-His and α-LdtJ antibodies. Protein concentrations were determined using the Bradford assay.

### Intact mass analysis of LdtJ

Purified wild-type LdtJ protein was separated on a 4–12% SDS-PAGE gel. The gel was stained with Coomassie Brilliant Blue for 10 min and destained overnight with gentle rocking. Protein bands corresponding to LdtJ were excised using a clean scalpel and transferred into 500 µL of dialysis buffer (10 mM Tris, 50 mM KCl, 0.1 mM EDTA, and 5% Glycerol, pH 8). Excised gel pieces were submitted to the Proteomics core facility of UT Southwestern Medical Center for intact mass spectrometry analysis.

### Enzymatic activity assays

Activity assays for wild-type (LdtJ) and the catalytic mutant (LdtJ_C390S_) were performed in a final reaction volume of 50 µL containing 20 mM Tris/HCl (pH 7.5), 100 mM NaCl and where indicated, 10 mM d-lysine. Each reaction included 10 µM of either LdtJ or LdtJ_C390S_. To initiate the reaction, 10 µL PG from *E. coli* BW25113Δ6LDT was added. Reactions were incubated overnight at 37°C. The enzymatic reaction was terminated by boiling for 10 min, followed by overnight digestion with cellosyl. After digestion, samples were again boiled for 10 min, then reduced with sodium borohydride and acidified to pH 4.0–4.5. As a control, PG incubated in pH 7.5 buffer without enzyme was processed in parallel. Muropeptide analysis was performed as described previously (65).

### Western blotting

Western blot analysis was done as previously described (66). Briefly, proteins were transferred to polyvinylidene difluoride membranes (Thermo Fisher Scientific) following SDS-PAGE. Membranes were blocked in 5% non-fat dry milk in Tris-buffered saline for 2 h at room temperature. Primary antibodies were used at the following dilutions: α-LdtJ (1:500), α-His (1:500), and α-RpoA (1:1,000), followed by HRP -conjugated α-rabbit-IgG for α-LdtJ and α-RpoA and HRP -conjugated α-mouse for α-His at 1:10,000 (Thermo Fisher Scientific). LdtJ antisera was generated in rabbits using the C-terminal peptide sequence DANDLGNKVRSGVTVKFLE. Signal detection was performed using SuperSignal West Pico Plus Chemiluminescent Substrate (Thermo Fisher Scientific).

### RNA sequencing

Transcriptomic analysis was performed as previously described, with modification (67–70). Total RNA was extracted from *A. baumannii* ATCC 17978 cultures grown in triplicates using the Direct-Zol RNA miniprep kit (Zymo Research), following the manufacturer’s protocol. RNA samples were submitted to the SeqCenter for Illumina RNA sequencing. The resulting reads were aligned to the *A. baumannii* ATCC 17978 reference genome using CLC genomic workbench software (Qiagen). Gene expression levels were quantified as reads per kilobase per million mapped reads (RPKM) and weighted-proportions fold changes were calculated to compare expression across strains. Baggerley’s test on proportions was used to assess differential expression, and *p*-values were calculated by a two-tailed *t*-test to determine statistical significance. The data were plotted in GraphPad Prism 10 (version 8.4.1). Data accession number: GSE304602.

### Reverse transcriptase quantitative PCR

RT-qPCR was done as previously reported (68). Briefly, overnight cultures were back-diluted to OD_600_ of 0.05 and grown at 37°C in LB broth until reaching mid-logarithmic phase. Total RNA was extracted using the Direct-zol RNA Miniprep Kit (Zymo Research) according to the manufacturer’s instructions. Genomic DNA contamination was removed using the TURBO DNA-free Kit (Thermo Fisher Scientific). For each strain, 500 ng of DNase-treated RNA was used for cDNA synthesis using random hexamers and the SuperScript III First-Strand Synthesis System (Thermo Fisher Scientific). Quantitative PCR reactions were assembled with 2 µL of cDNA, 0.2 µM gene-specific primers (listed in Table S5; designed using Primer3Plus, Andreas Untergasser), and PowerUp SYBR Green Master Mix (Thermo Fisher Scientific). Reactions were run in technical triplicates using the QuantStudio 3 Real-Time PCR System (Applied Biosystems). The housekeeping gene *rpoA* was used as an endogenous control. No-reverse transcriptase controls were included to confirm the absence of genomic DNA contamination. Relative fold change (Rf) values were calculated using the comparative Ct method, and data were analyzed using DA2 software (Thermo Fisher Scientific); manual ΔΔCt calculations were not performed.

### Radiolabeled extraction and detection of (p)ppGpp

Radiolabeled pp(G)pp extraction and detection were performed as previously described (43), with minor modifications. Overnight cultures were back-diluted to OD_600_ 0.05 in LB broth supplemented with 5 µCi/mL ^32^P and incubated at 37°C with shaking for 3 h. Cultures were then normalized to the lowest OD_600_ value across samples. For each sample, 500 µL of culture was pelleted and resuspended in 100 µL of 2 N Formic Acid. Samples were incubated at room temperature for 15 min and then frozen at −80°C for 15 min. This freeze-thaw cycle was repeated once more. After centrifugation to remove cellular debris, the supernatant was collected. 10 µL of each sample was spotted on a PEI-Cellulose TLC plate and resolved using a 1.5 M potassium dihydrogen phosphate (KH_2_PO_4_), pH 3.4 solvent system. The TLC plate was exposed to a phosphor imaging screen for 48 h, and signals were visualized using a Storm Molecular Imaging scanner. Spot intensities were quantified using ImageJ (Fiji), and data were plotted using GraphPad Prism 10 (version 8.4.1) software.

## SUPPLEMENTAL MATERIAL

Supplemental figures and tables jb.00331-25-s0001.docx Fig. S1: Purification of LdtJ and LdtJC390S. Fig. S2: Disruption of outer membrane (OM) asymmetry restores growth fitness in the ΔldtJ mutant. Table S4: Strains and plasmids used in this study. Table S5: Primers used in this study.

Table S1 jb.00331-25-s0002.xlsx Differential gene expression WT vs. Δ*ldtJ*.

Table S2 jb.00331-25-s0003.xlsx Differential gene expression Δ*ldtJ* vs. Δ*ldtJ* Δ*mla*.

Table S3 jb.00331-25-s0004.xlsx Differential gene expression WT vs. Δ*ldtJ* Δ*mla*.

ASM does not own the copyrights to Supplemental Material that may be linked to, or accessed through, an article. The authors have granted ASM a non-exclusive, world-wide license to publish the Supplemental Material files. Please contact the corresponding author directly for reuse.

## References

[1] Typas A, Banzhaf M, Gross CA, Vollmer W. 2011. From the regulation of peptidoglycan synthesis to bacterial growth and morphology. Nat Rev Microbiol 10:123–136. doi:10.1038/nrmicro2677 22203377 PMC5433867

[2] Irazoki O, Hernandez SB, Cava F. 2019. Peptidoglycan muropeptides: release, perception, and functions as signaling molecules. Front Microbiol 10:500. doi:10.3389/fmicb.2019.00500 30984120 PMC6448482

[3] Vollmer W, Blanot D, de Pedro MA. 2008. Peptidoglycan structure and architecture. FEMS Microbiol Rev 32:149–167. doi:10.1111/j.1574-6976.2007.00094.x 18194336

[4] Turner RD, Vollmer W, Foster SJ. 2014. Different walls for rods and balls: the diversity of peptidoglycan. Mol Microbiol 91:862–874. doi:10.1111/mmi.12513 24405365 PMC4015370

[5] Garde S, Chodisetti PK, Reddy M. 2021. Peptidoglycan: structure, synthesis, and regulation. EcoSal Plus 9:eESP-0010-2020. doi:10.1128/ecosalplus.ESP-0010-2020 PMC11168573 33470191

[6] Vollmer W, Bertsche U. 2008. Murein (peptidoglycan) structure, architecture and biosynthesis in Escherichia coli. Biochim Biophys Acta 1778:1714–1734. doi:10.1016/j.bbamem.2007.06.007 17658458

[7] Alvarez L, Hernandez SB, Torrens G, Weaver AI, Dörr T, Cava F. 2024. Control of bacterial cell wall autolysins by peptidoglycan crosslinking mode. Nat Commun 15:7937. doi:10.1038/s41467-024-52325-2 39261529 PMC11390936

[8] Lee TK, Tropini C, Hsin J, Desmarais SM, Ursell TS, Gong E, Gitai Z, Monds RD, Huang KC. 2014. A dynamically assembled cell wall synthesis machinery buffers cell growth. Proc Natl Acad Sci USA 111:4554–4559. doi:10.1073/pnas.1313826111 24550500 PMC3970539

[9] Egan AJF, Errington J, Vollmer W. 2020. Regulation of peptidoglycan synthesis and remodelling. Nat Rev Microbiol 18:446–460. doi:10.1038/s41579-020-0366-3 32424210

[10] Schleifer KH, Kandler O. 1972. Peptidoglycan types of bacterial cell walls and their taxonomic implications. Bacteriol Rev 36:407–477. doi:10.1128/br.36.4.407-477.1972 4568761 PMC408328

[11] Espaillat A, Alvarez L, Torrens G, Ter Beek J, Miguel-Ruano V, Irazoki O, Gago F, Hermoso JA, Berntsson RP-A, Cava F. 2024. A distinctive family of L,D-transpeptidases catalyzing L-Ala-mDAP crosslinks in Alpha- and Betaproteobacteria. Nat Commun 15:1343. doi:10.1038/s41467-024-45620-5 38351082 PMC10864386

[12] Aliashkevich A, Cava F. 2022. LD-transpeptidases: the great unknown among the peptidoglycan cross-linkers. FEBS J 289:4718–4730. doi:10.1111/febs.16066 34109739

[13] Sanders AN, Pavelka MS. 2013. Phenotypic analysis of Eschericia coli mutants lacking L,D-transpeptidases. Microbiology (Reading) 159:1842–1852. doi:10.1099/mic.0.069211-0 23832002 PMC3783015

[14] Glauner B, Höltje JV, Schwarz U. 1988. The composition of the murein of Escherichia coli. J Biol Chem 263:10088–10095. doi:10.1016/S0021-9258(19)81481-3 3292521

[15] Morè N, Martorana AM, Biboy J, Otten C, Winkle M, Serrano CKG, Montón Silva A, Atkinson L, Yau H, Breukink E, den Blaauwen T, Vollmer W, Polissi A. 2019. Peptidoglycan remodeling enables Escherichia coli to survive severe outer membrane assembly defect. mBio 10:e02729-18. doi:10.1128/mBio.02729-18 30723128 PMC6428754

[16] Kuru E, Radkov A, Meng X, Egan A, Alvarez L, Dowson A, Booher G, Breukink E, Roper DI, Cava F, Vollmer W, Brun Y, VanNieuwenhze MS. 2019. Mechanisms of incorporation for D-Amino acid probes that target peptidoglycan biosynthesis. ACS Chem Biol 14:2745–2756. doi:10.1021/acschembio.9b00664 31743648 PMC6929685

[17] Caveney NA, Caballero G, Voedts H, Niciforovic A, Worrall LJ, Vuckovic M, Fonvielle M, Hugonnet J-E, Arthur M, Strynadka NCJ. 2019. Structural insight into YcbB-mediated beta-lactam resistance in Escherichia coli. Nat Commun 10:1849. doi:10.1038/s41467-019-09507-0 31015395 PMC6478713

[18] Peters K, Pazos M, Edoo Z, Hugonnet JE, Martorana AM, Polissi A, VanNieuwenhze MS, Arthur M, Vollmer W. 2018. Copper inhibits peptidoglycan LD-transpeptidases suppressing β-lactam resistance due to bypass of penicillin-binding proteins. Proc Natl Acad Sci USA 115:10786–10791. doi:10.1073/pnas.1809285115 30275297 PMC6196517

[19] Nallamotu KC, Bahadur R, Kaul M, Reddy M. 2023. Peptidoglycan remodeling by an L,D-transpeptidase, LdtD during cold shock in Escherichia coli. J Bacteriol 205:e0038222. doi:10.1128/jb.00382-22 36507682 PMC9879098

[20] Islam N, Kazi MI, Kang KN, Biboy J, Gray J, Ahmed F, Schargel RD, Boutte CC, Dörr T, Vollmer W, Boll JM. 2022. Peptidoglycan recycling promotes outer membrane integrity and carbapenem tolerance in Acinetobacter baumannii. MBio 13:e0100122. doi:10.1128/mbio.01001-22 35638738 PMC9239154

[21] Kang KN, Kazi MI, Biboy J, Gray J, Bovermann H, Ausman J, Boutte CC, Vollmer W, Boll JM. 2021. Septal class A penicillin-binding protein activity and ld-transpeptidases mediate selection of colistin-resistant lipooligosaccharide-deficient Acinetobacter baumannii. mBio 12:e02185-20. doi:10.1128/mBio.02185-20 33402533 PMC8545086

[22] Malinverni JC, Silhavy TJ. 2009. An ABC transport system that maintains lipid asymmetry in the gram-negative outer membrane. Proc Natl Acad Sci USA 106:8009–8014. doi:10.1073/pnas.0903229106 19383799 PMC2683108

[23] Kaur M, Mingeot-Leclercq MP. 2024. Maintenance of bacterial outer membrane lipid asymmetry: insight into MlaA. BMC Microbiol 24:186. doi:10.1186/s12866-023-03138-8 38802775 PMC11131202

[24] Shrivastava R, Jiang X, Chng SS. 2017. Outer membrane lipid homeostasis via retrograde phospholipid transport in Escherichia coli. Mol Microbiol 106:395–408. doi:10.1111/mmi.13772 28815827

[25] Powers MJ, Trent MS. 2018. Phospholipid retention in the absence of asymmetry strengthens the outer membrane permeability barrier to last-resort antibiotics. Proc Natl Acad Sci USA 115:E8518–E8527. doi:10.1073/pnas.1806714115 30087182 PMC6130378

[26] Chong ZS, Woo WF, Chng SS. 2015. Osmoporin OmpC forms a complex with MlaA to maintain outer membrane lipid asymmetry in Escherichia coli. Mol Microbiol 98:1133–1146. doi:10.1111/mmi.13202 26314242

[27] Ekiert DC, Bhabha G, Isom GL, Greenan G, Ovchinnikov S, Henderson IR, Cox JS, Vale RD. 2017. Architectures of lipid transport systems for the bacterial outer membrane. Cell 169:273–285. doi:10.1016/j.cell.2017.03.019 28388411 PMC5467742

[28] Thong S, Ercan B, Torta F, Fong ZY, Wong HYA, Wenk MR, Chng SS. 2016. Defining key roles for auxiliary proteins in an ABC transporter that maintains bacterial outer membrane lipid asymmetry. elife 5:e19042. doi:10.7554/eLife.19042 27529189 PMC5016091

[29] Kamischke C, Fan J, Bergeron J, Kulasekara HD, Dalebroux ZD, Burrell A, Kollman JM, Miller SI. 2019. The Acinetobacter baumannii Mla system and glycerophospholipid transport to the outer membrane. elife 8:e40171. doi:10.7554/eLife.40171 30638443 PMC6365058

[30] Yeow J, Luo M, Chng SS. 2023. Molecular mechanism of phospholipid transport at the bacterial outer membrane interface. Nat Commun 14:8285. doi:10.1038/s41467-023-44144-8 38092770 PMC10719372

[31] Mueller EA, Levin PA. 2020. Bacterial cell wall quality control during environmental stress. mBio 11:e02456-20. doi:10.1128/mBio.02456-20 33051371 PMC7554673

[32] Egan AJF, Vollmer W. 2013. The physiology of bacterial cell division. Ann N Y Acad Sci 1277:8–28. doi:10.1111/j.1749-6632.2012.06818.x 23215820

[33] Rojas ER, Billings G, Odermatt PD, Auer GK, Zhu L, Miguel A, Chang F, Weibel DB, Theriot JA, Huang KC. 2018. The outer membrane is an essential load-bearing element in Gram-negative bacteria. Nature 559:617–621. doi:10.1038/s41586-018-0344-3 30022160 PMC6089221

[34] Sun J, Rutherford ST, Silhavy TJ, Huang KC. 2022. Physical properties of the bacterial outer membrane. Nat Rev Microbiol 20:236–248. doi:10.1038/s41579-021-00638-0 34732874 PMC8934262

[35] Paetzel M, Karla A, Strynadka NCJ, Dalbey RE. 2002. Signal peptidases. Chem Rev 102:4549–4580. doi:10.1021/cr010166y 12475201

[36] Auclair SM, Bhanu MK, Kendall DA. 2012. Signal peptidase I: cleaving the way to mature proteins. Protein Sci 21:13–25. doi:10.1002/pro.757 22031009 PMC3323777

[37] Kohn WD, Kay CM, Hodges RS. 1997. Salt effects on protein stability: two-stranded alpha-helical coiled-coils containing inter- or intrahelical ion pairs. J Mol Biol 267:1039–1052. doi:10.1006/jmbi.1997.0930 9135129

[38] Kazi MI, Schargel RD, Boll JM. 2020. Generating transposon insertion libraries in gram-negative bacteria for high-throughput sequencing. J Vis Exp 161. doi:10.3791/61612 PMC7861349 32716393

[39] Walsh CT. 1989. Enzymes in the D-alanine branch of bacterial cell wall peptidoglycan assembly. J Biol Chem 264:2393–2396. doi:10.1016/S0021-9258(19)81624-1 2644260

[40] Cava F, Lam H, de Pedro MA, Waldor MK. 2011. Emerging knowledge of regulatory roles of D-amino acids in bacteria. Cell Mol Life Sci 68:817–831. doi:10.1007/s00018-010-0571-8 21161322 PMC3037491

[41] Nandineni MR, Gowrishankar J. 2004. Evidence for an arginine exporter encoded by yggA (argO) that is regulated by the LysR-type transcriptional regulator ArgP in Escherichia coli. J Bacteriol 186:3539–3546. doi:10.1128/JB.186.11.3539-3546.2004 15150242 PMC415761

[42] Potrykus K, Cashel M. 2008. (P)ppGpp: still magical? Annu Rev Microbiol 62:35–51. doi:10.1146/annurev.micro.62.081307.162903 18454629

[43] Powers MJ, Simpson BW, Trent MS. 2020. The Mla pathway in Acinetobacter baumannii has no demonstrable role in anterograde lipid transport. elife 9:e56571. doi:10.7554/eLife.56571 32880370 PMC7500953

[44] Magnet S, Bellais S, Dubost L, Fourgeaud M, Mainardi JL, Petit-Frère S, Marie A, Mengin-Lecreulx D, Arthur M, Gutmann L. 2007. Identification of the L,D-transpeptidases responsible for attachment of the Braun lipoprotein to Escherichia coli peptidoglycan. J Bacteriol 189:3927–3931. doi:10.1128/JB.00084-07 17369299 PMC1913343

[45] Lavollay M, Arthur M, Fourgeaud M, Dubost L, Marie A, Veziris N, Blanot D, Gutmann L, Mainardi J-L. 2008. The peptidoglycan of stationary-phase Mycobacterium tuberculosis predominantly contains cross-links generated by L,D-transpeptidation. J Bacteriol 190:4360–4366. doi:10.1128/JB.00239-08 18408028 PMC2446752

[46] Cordillot M, Dubée V, Triboulet S, Dubost L, Marie A, Hugonnet JE, Arthur M, Mainardi JL. 2013. In vitro cross-linking of Mycobacterium tuberculosis peptidoglycan by L,D-transpeptidases and inactivation of these enzymes by carbapenems. Antimicrob Agents Chemother 57:5940–5945. doi:10.1128/AAC.01663-13 24041897 PMC3837840

[47] Zellner B, Mengin-Lecreulx D, Tully B, Gunning WT 3rd, Booth R, Huntley JF. 2021. A Francisella tularensis L,D-carboxypeptidase plays important roles in cell morphology, envelope integrity, and virulence. Mol Microbiol 115:1357–1378. doi:10.1111/mmi.14685 33469978

[48] Frirdich E, Vermeulen J, Biboy J, Soares F, Taveirne ME, Johnson JG, DiRita VJ, Girardin SE, Vollmer W, Gaynor EC. 2014. Peptidoglycan ld-Carboxypeptidase Pgp2 influences campylobacter jejuni helical cell shape and pathogenic properties and provides the substrate for the dl-Carboxypeptidase Pgp1. J Biol Chem 289:8007–8018. doi:10.1074/jbc.M113.491829 24394413 PMC3961634

[49] García-Heredia A, Pohane AA, Melzer ES, Carr CR, Fiolek TJ, Rundell SR, Lim HC, Wagner JC, Morita YS, Swarts BM, Siegrist MS. 2018. Peptidoglycan precursor synthesis along the sidewall of pole-growing mycobacteria. elife 7:e37243. doi:10.7554/eLife.37243 30198841 PMC6191288

[50] Dörr T, Cava F, Lam H, Davis BM, Waldor MK. 2013. Substrate specificity of an elongation-specific peptidoglycan endopeptidase and its implications for cell wall architecture and growth of Vibrio cholerae. Mol Microbiol 89:949–962. doi:10.1111/mmi.12323 23834664 PMC3769093

[51] Konovalova A, Kahne DE, Silhavy TJ. 2017. Outer membrane biogenesis. Annu Rev Microbiol 71:539–556. doi:10.1146/annurev-micro-090816-093754 28886680 PMC5778897

[52] Hauryliuk V, Atkinson GC, Murakami KS, Tenson T, Gerdes K. 2015. Recent functional insights into the role of (p)ppGpp in bacterial physiology. Nat Rev Microbiol 13:298–309. doi:10.1038/nrmicro3448 25853779 PMC4659695

[53] Potrykus K, Murphy H, Philippe N, Cashel M. 2011. ppGpp is the major source of growth rate control in E. coli. Environ Microbiol 13:563–575. doi:10.1111/j.1462-2920.2010.02357.x 20946586 PMC4556285

[54] Büke F, Grilli J, Cosentino Lagomarsino M, Bokinsky G, Tans SJ. 2022. ppGpp is a bacterial cell size regulator. Curr Biol 32:870–877. doi:10.1016/j.cub.2021.12.033 34990598

[55] Zhao H, Roistacher DM, Helmann JD. 2018. Aspartate deficiency limits peptidoglycan synthesis and sensitizes cells to antibiotics targeting cell wall synthesis in Bacillus subtilis. Mol Microbiol 109:826–844. doi:10.1111/mmi.14078 29995990 PMC6185803

[56] Liu F, Qimuge S, Hao J, Yan H, Bach T, Fan L, Morigen S. 2014. AspC-mediated aspartate metabolism coordinates the Escherichia coli cell cycle. PLoS One 9:e92229. doi:10.1371/journal.pone.0092229 24670900 PMC3966765

[57] Wood JM. 1981. Genetics of L-proline utilization in Escherichia coli. J Bacteriol 146:895–901. doi:10.1128/jb.146.3.895-901.1981 7016835 PMC216941

[58] Groisman EA, Hollands K, Kriner MA, Lee EJ, Park SY, Pontes MH. 2013. Bacterial Mg2+ homeostasis, transport, and virulence. Annu Rev Genet 47:625–646. doi:10.1146/annurev-genet-051313-051025 24079267 PMC4059682

[59] Ikai H, Yamamoto S. 1997. Identification and analysis of a gene encoding L-2,4-diaminobutyrate:2-ketoglutarate 4-aminotransferase involved in the 1,3-diaminopropane production pathway in Acinetobacter baumannii. J Bacteriol 179:5118–5125. doi:10.1128/jb.179.16.5118-5125.1997 9260954 PMC179370

[60] Yamamoto S, Tsuzaki Y, Tougou K, Shinoda S. 1992. Purification and characterization of L-2,4-diaminobutyrate decarboxylase from Acinetobacter calcoaceticus. J Gen Microbiol 138:1461–1465. doi:10.1099/00221287-138-7-1461 1512577

[61] Höltje JV. 1996. Bacterial lysozymes. EXS 75:65–74. doi:10.1007/978-3-0348-9225-4_4 8765294

[62] Noel HR, Keerthi S, Ren X, Winkelman JD, Troutman JM, Palmer LD. 2024. Genetic synergy between Acinetobacter baumannii undecaprenyl phosphate biosynthesis and the Mla system impacts cell envelope and antimicrobial resistance . mBio 15:e0280423. doi:10.1128/mbio.02804-23 38364179 PMC10936186

[63] Mikheyeva IV, Sun J, Huang KC, Silhavy TJ. 2023. Mechanism of outer membrane destabilization by global reduction of protein content. Nat Commun 14:5715. doi:10.1038/s41467-023-40396-6 37714857 PMC10504340

[64] Tucker AT, Nowicki EM, Boll JM, Knauf GA, Burdis NC, Trent MS, Davies BW. 2014. Defining gene-phenotype relationships in Acinetobacter baumannii through one-step chromosomal gene inactivation. mBio 5:e01313-14. doi:10.1128/mBio.01313-14 25096877 PMC4128354

[65] Glauner B. 1988. Separation and quantification of muropeptides with high-performance liquid chromatography. Anal Biochem 172:451–464. doi:10.1016/0003-2697(88)90468-x 3056100

[66] Kang K.N, Boll JM. 2022. PBP1A directly interacts with the divisome complex to promote septal peptidoglycan synthesis in Acinetobacter baumannii. J Bacteriol 204:e0023922. doi:10.1128/jb.00239-22 36317921 PMC9765026

[67] Boll JM, Crofts AA, Peters K, Cattoir V, Vollmer W, Davies BW, Trent MS. 2016. A penicillin-binding protein inhibits selection of colistin-resistant, lipooligosaccharide-deficient Acinetobacter baumannii. Proc Natl Acad Sci USA 113:E6228–E6237. doi:10.1073/pnas.1611594113 27681618 PMC5068286

[68] Olea-Ozuna RJ, Campbell MJ, Quintanilla SY, Nandy S, Brodbelt JS, Boll JM. 2025. Alternative lipid synthesis in response to phosphate limitation promotes antibiotic tolerance in Gram-negative ESKAPE pathogens. PLoS Pathog 21:e1012933. doi:10.1371/journal.ppat.1012933 39919117 PMC11828411

[69] Murtha AN, Kazi MI, Schargel RD, Cross T, Fihn C, Cattoir V, Carlson EE, Boll JM, Dörr T. 2022. High-level carbapenem tolerance requires antibiotic-induced outer membrane modifications. PLoS Pathog 18:e1010307. doi:10.1371/journal.ppat.1010307 35130322 PMC8853513

[70] Kang Katie N, Klein DR, Kazi MI, Guérin F, Cattoir V, Brodbelt JS, Boll JM. 2019. Colistin heteroresistance in Enterobacter cloacae is regulated by PhoPQ-dependent 4-amino-4-deoxy-l-arabinose addition to lipid A. Mol Microbiol 111:1604–1616. doi:10.1111/mmi.14240 30873646 PMC6561824

