# Supplementary material for: Molecular interplay between peptidoglycan integrity and outer membrane asymmetry in maintaining cell envelope homeostasis
Source: J Bacteriol. 2025 Sep 18;207(10):e00331-25. doi: 10.1128/jb.00331-25 (PMC12548408; doi:10.1128/jb.00331-25)
Supplement: Supplemental figures and tables — Fig. S1: Purification of LdtJ and LdtJC390S. Fig. S2: Disruption of outer membrane (OM) asymmetry restores growth fitness in the ΔldtJ mutant. Table S4: Strains and plasmids used in this study. Table S5: Primers used in this study. [file jb.00331-25-s0001.docx]

**Supplemental data for ‘Molecular interplay between peptidoglycan integrity and outer membrane asymmetry in maintaining cell envelope homeostasis*’***

Sinjini Nandy^1^, Arshya F. Tehrani^1^, Augusto C. Hunt-Serracin^1^, Jacob Biboy^2^, Christine Pybus^1^, Waldemar Vollmer^2,3^, Joseph M. Boll^1*^


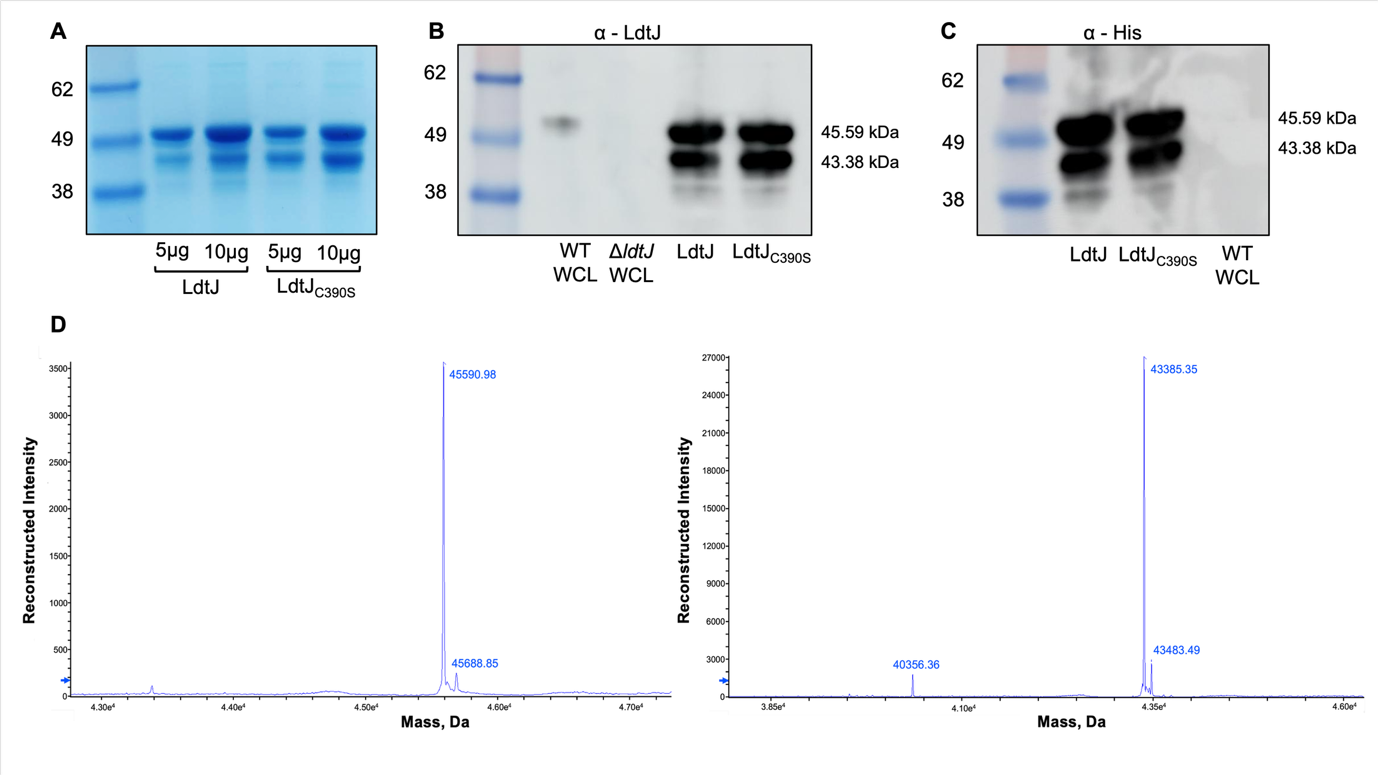
**Supplementary Figures**

**Figure S1. Purification of LdtJ and LdtJ_C390S_. (A)** Coomassie stained SDS-PAGE gel showing purified recombinant LdtJ and the catalytically inactive mutant LdtJ_C390S_. **(B)** Western blot using α-LdtJ antibody and **(C)** α-His antibody. (WCL = Whole cell lysate) **(D)** Mass spectrometry analysis of purified LdtJ revealed two major products with molecular weights of 45.59 kDa and 43.38 kDa, consistent with the bands observed in panels A–C. Each experiment was independently replicated three times, and representative results are shown.

***
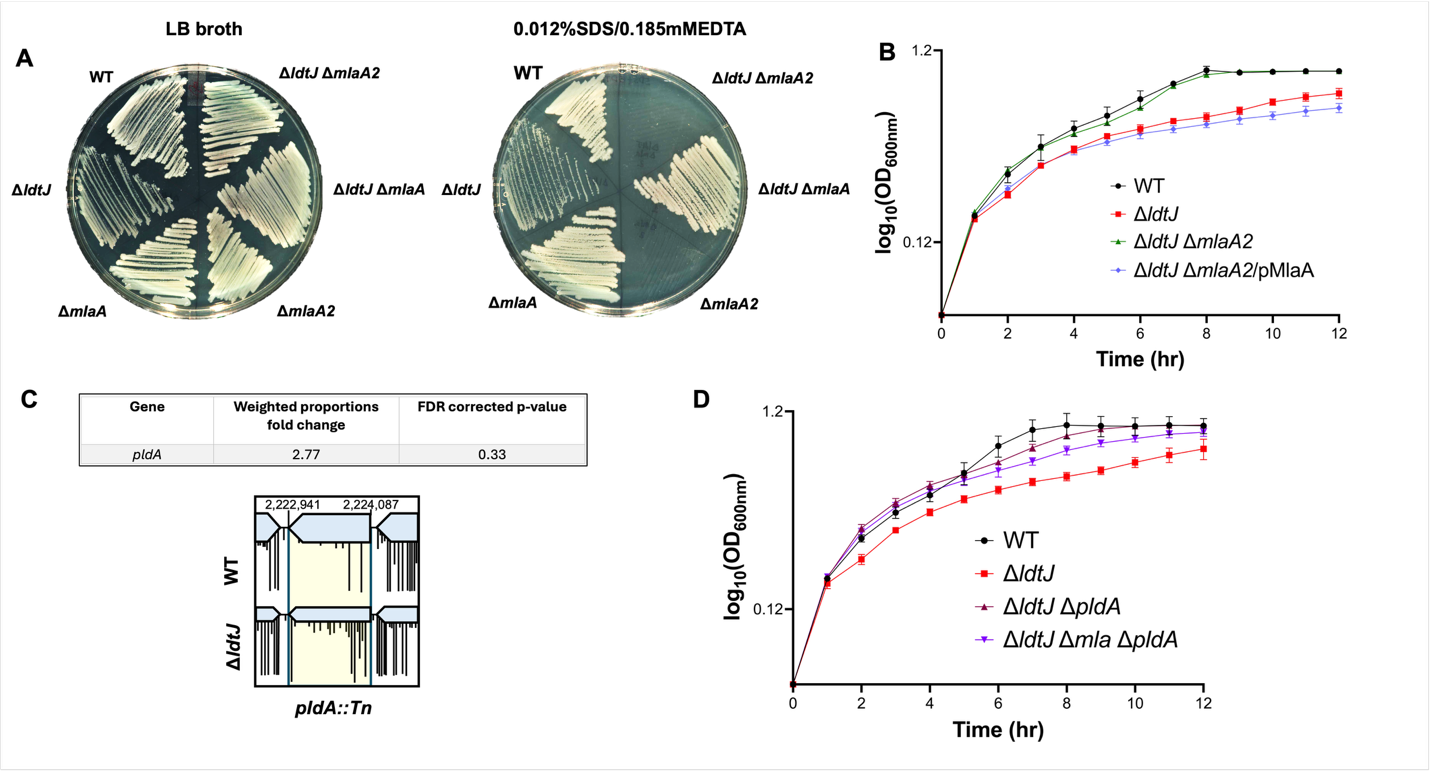
***

**Figure S2. Disruption of outer membrane (OM) asymmetry restores growth fitness in the Δ*ldtJ* mutant. (A)** SDS/EDTA sensitivity assay of strains with Mla pathway disruption, showing both SDS/EDTA-resistant and -sensitive replicates. **(B)** Growth curve analysis of the SDS/EDTA-sensitive Δ*ldtJ* Δ*mlaA2* strain, demonstrating restored growth fitness like the SDS/EDTA-resistant Δ*ldtJ* Δ*mlaA* strain. **(C)** Transposon sequencing data and insertion profiles for *pldA::Tn* in WT and Δ*ldtJ* backgrounds, indicating significant fold changes in the *pldA* system. **(D)** Growth curve analysis of WT, Δ*ldtJ*, Δ*ldtJ* Δ*pldA*, and Δ*ldtJ* Δ*mla* Δ*pldA* strains. All experiments were independently replicated three times; representative datasets are shown.

**Supplementary Tables**

**Tables S1-3: Please See the Excel Spreadsheets**

**Table S4: Strains and plasmids used in this study.**

| **Strain or Plasmid** | **Description** | **Reference or Source** |
| --- | --- | --- |
| **Strains** |  |  |
| ***A. baumannii* ATCC 17978** | Wild type | [1] |
| ***A. baumannii* ATCC 17978** | *ldtJ* (*A1S_2371*) | [2] |
| ***A. baumannii* ATCC 17978** | *ldtJ mlaA* | This study |
| ***A. baumannii* ATCC 17978** | *ldtJ mlaE* | This study |
| ***A. baumannii* ATCC 17978** | *ldtJ pldA* | This study |
| ***A. baumannii* ATCC 17978** | *ldtJ mla pldA* | This study |
| ***A. baumannii* ATCC 17978** | *mlaA* (*A1S_0622*) | This study |
| ***A. baumannii* ATCC 17978** | *mlaE* (*A1S_3102*) | This study |
| ***E. coli* DH5** | Host strain for cloning | [3] |
| **Plasmids** |  |  |
| pAT03 | pMMB67EH with FLP recombinase, Tet^R^ | [4] |
| pAT04 | pMMB67EH with Rec_AB_ system, Tet^R^ | [4] |
| pKD4 | Kan^R^ | [5] |
| pMMB67EH | pMMB67EH with the Kan^R^ gene from pKD4 inserted into the PvuI site, Kan^R^ | [2] |
| pLdtJ | pMMB67EHKn carrying *ldtJ* (*A1S_2371*) | [2] |
| **pLdtJ_C390S_** | pMMB67EHKn::*ldtJ_C390S_* | This study |
| **pLdtJ_40-146_** | pMMB67EHKn::*ldtJ_40-146_* | This study |
| **pLdtJ_147-192_** | pMMB67EHKn::*ldtJ_147-192_* | This study |
| **pLdtJ_193-282_** | pMMB67EHKn::*ldtJ_193-282_* | This study |
| **pLdtJ_YkuD_** | pMMB67EHKn::*ldtJ_YkuD_* | This study |
| pMlaA | pMMB67EHKn carrying *mlaA* (*A1S_0622*) | This study |

**Table S5: Primers used in this study.**

| **Oligo Name** | **Sequence (5’-3’)** |
| --- | --- |
| **Deletion Primers** |  |
| ***mlaA* (A1S_0622) Kan-FRT 5’** | CAACGATATTTCTATATAATTTGATCAATTTTACATTGATAATA  GCTTATCGCTTCTGTATTAAAATATATCTCTACCCAACTGCATA  TTGAATATAGCCTGGCTAGCGGTTAAGGAATTATATGAGCGAT  TGTGTAGGCTGGAGCTGCTTCG |
| ***mlaA* (A1S_0622) Kan-FRT 3’** | TTATCAGTGTTATCTTCTGGTACATCTTCAGATTCGTCATCATC  AATAAAAGACACATCTGCCGAATCACCTTTTTTCTCGGCAAT  CTGGAATGCTTTACGTTGGAGATATAAATCACGAATCATATCC  TCCTTAGTTCCTATTCCG |
| ***mlaA* confirm 5’** | GATCGACTTTACATCTTTGGCACTGC |
| ***mlaA* confirm 3’** | CTGGTAATGCTTCCAGAGCTTGCT |
| ***mlaE* (A1S_3102) Kan-FRT 5’** | TCTATCAAATGCATCGTGTAGGGGTAATGTCTTACTCATTATCA  CGGTATCAGGTTTATTTATTGGTCTGGTACTCGGATTGCAAGGC  TACTCAATATTAGTCAATGTTGGTAGTGAATCAATGAGCGAT TGTGTAGGCTGGAGCTGCTTCG |
| ***mlaE* (A1S_3102) Kan-FRT 3’** | ACAACCGTGCGGGTCATTGCCGTTGCAATGCCTTCAGGTGTCG  GATCACATGCATACCCTTGGAAAACAGCAATCCATGTACACAG  CAAAGCAAATACAATGCTCTTAATAATGCCATTTACGACATATCC TCCTTAGTTCCTATTCCG |
| ***mlaE* confirm 5’** | GCGCTTGATCCCGATTTAATTATGTATGACG |
| ***mlaE* confirm 3’** | GCGTATAATCAGAACTATAACGTAATTCGTCTAAAGC |
| ***pldA* (A1S_1919) Kan-FRT 5’** | CAATATTTAATTGAACAAGCACCTGTAAAAGCTGTCGTTGCAC  CTTTTGCAAAACGTGATGAATTGCAACAACTGGGTTTTACGAT  CAAACAAGTTAATTAAAATAAAAATTGGAGATGAACATGAGC  GATTGTGTAGGCTGGAGCTGCTTCG |
| ***pldA* (A1S_1919) Kan-FRT 3’** | CCTTTAACACTCGCCTATTTAATTGCTACTCCTAAAAAAAGCC  GTCTCAAACATCCTCAAGCCAACATTCAAATTGGTTAATAAAA  AAGCCGCTGTAAAAGCGGCATTTTTATAGGTCAAACTTAATAT  CCTCCTTAGTTCCTATTCCG |
| ***pldA* confirm 5’** | GGTGGCAAGTATGGACAAACCTG |
| ***pldA* confirm 3’** | CCTGTACAGTAGGGCTTTCGCG |
| ***ldtJ* (A1S_2371) Kan-FRT 5’** | TTATATCCCTTCGCGTCTCAAATAAGCCAATATTAAATTCATAA  GAATGAATGATTGGTGAGTTTATGGCCTAAAGGATCTGATTTT  CCCTATTGCTTATATGAAAATTCTTAAGGTTGAATTACAGCGAT  TGTGTAGGCTGGAGCTGCTTCG |
| ***ldtJ* (A1S_2371) Kan-FRT 3’** | TTAGTAAACCTAGGCTGGTTTTATTTTTATAATCAAAACAATAA  CTACATATTCCACGGGGCTATGCTAAAAAATTTAATAAAAAAG  CCTGCATAAAGCAGGCTCTTTTAATTAAGAGGAATATCCTCCT  TAGTTCCTATTCCG |
| ***ldtJ* confirm 5’** | TACTTGCAGCATGTTACATCGGGTTTA |
| ***ldtJ* confirm 3’** | GGGTCAGATGCTGAAGCTGAATGGTTA |
| **Complementation Primers** |  |
| ***mlaA* EcoRI 5’** | CGCGAATTCATGAATTATTCTAATTTACTTTTGTCGAGCTTATTA  ACTGTAGGTC |
| ***mlaA* KpnI 3’** | CGCGGTACCTTATTTTTCGGTTTTATCAGTGTTATCTTCTGGTAC  ATCTT |
| ***ldtJ* KpnI 5’** | CGCGGTACCATGTTTGTTCGCTCATTACTCGCTATGAGTTTAAG  TTGTATTATTGCTAATGTTGCTTTGGCTGCG |
| ***ldtJ_C390S_* SalI 3’** | CGCGTCGACTTATTCTAAGAATTTAACAGTTACGCCTGAACGTA  CTTTATTACCTAAATCGTTAGCATCCCAGTTCGTTAAACGGAT  ACTACC |
| pMMB67EH confirm 5’ | CGGTTCTGGCAAATATTCTGAAA |
| pMMB67EH confirm 3’ | CTGCGTTCTGATTTAATCTGTAT |
| **Purification primers** |  |
| ***ldtJ* NdeI 5’** | CGCCATATGTTTGTTCGCTCATTACTCGC |
| ***ldtJ* His 8X BamHI 3’** | CGCGGATCCTTAATGGTGATGGTGATGGTGATGGTGTTCTAA  GAATTTAACAGTTA |
| ***ldtJ_C390S_* His 8X BamHI 3’** | CGCGGATCCTTAATGGTGATGGTGATGGTGATGGTGTTCTAA  GAATTTAACAGTTACGCCTGAACGTACTTTATTACCTAAATC  GTTAGCATCCCAGTTCGTTAAACGGATACTACCGTG |
| pT7-7 confirm 5’ | CGATTCGAACTTCTGATA |
| pT7-7 confirm 3’ | ATCGATGATAAGCTT |
| **qPCR primers** |  |
| ***A1S_0095* 5’** | CGGGTATTCACTACGAAAACCG |
| ***A1S_0095* 3’** | TGAACCGCTTCCATTTGTGC |
| ***A1S_0096* 5’** | AGCGCAAATTGAGTGGGTAG |
| ***A1S_0096* 3’** | TCCCAAGCCTGCACATAATG |
| ***A1S_1046* 5’** | TGCCGCGTTTAAAACTACCC |
| ***A1S_1046* 3’** | CGCAACCGAACCAATCAGTAC |
| ***rpoA* 5’** | AATGCGCGTGTAGAACAACG |
| ***rpoA* 3’** | CAAGATTGTTGCCGCTTTGC |

**REFERENCES**

1. Baumann P, Doudoroff M, Stanier RY. A study of the Moraxella group. II. Oxidative-negative species (genus *Acinetobacter*). *J Bacteriol*. 1968 May;95(5):1520–41.

2. Kang KN, Kazi MI, Biboy J, Gray J, Bovermann H, Ausman J, et al. Septal Class A Penicillin-Binding Protein Activity and ld-Transpeptidases Mediate Selection of Colistin-Resistant Lipooligosaccharide-Deficient *Acinetobacter baumannii*. *mBio*. 2021 Jan 5;12(1):e02185-20.

3. Hanahan D. Studies on transformation of *Escherichia coli* with plasmids. *J Mol Biol*. 1983 Jun 5;166(4):557–80.

4. Tucker AT, Nowicki EM, Boll JM, Knauf GA, Burdis NC, Trent MS, et al. Defining gene-phenotype relationships in *Acinetobacter baumannii* through one-step chromosomal gene inactivation. *mBio*. 2014 Aug 5;5(4):e01313-01314.

5. Datsenko KA, Wanner BL. One-step inactivation of chromosomal genes in *Escherichia coli* K-12 using PCR products. *Proc Natl Acad Sci U S A*. 2000 Jun 6;97(12):6640–5.
